# Dopamine regulates stimulus generalization in the human hippocampus

**DOI:** 10.7554/eLife.12678

**Published:** 2016-02-02

**Authors:** Thorsten Kahnt, Philippe N Tobler

**Affiliations:** 1 Department of Neurology, Northwestern University Feinberg School of Medicine, Chicago, United States; 2 Department of Economics, Laboratory for Social and Neural Systems Research, University of Zurich, Zürich, Switzerland; Brown University, United States

**Keywords:** associative learning, reward, fMRI, dopamine, hippocampus, Human

## Abstract

The ability to generalize previously learned information to novel situations is fundamental for adaptive behavior. However, too wide or too narrow generalization is linked to neuropsychiatric disorders. Previous research suggests that interactions between the dopaminergic system and the hippocampus may play a role in generalization, but whether and how the degree of generalization can be modulated via these pathways is currently unknown. Here, we addressed this question in humans using pharmacology, functional magnetic resonance imaging, and computational modeling. Blocking dopamine D2-receptors (D2R) altered generalization behavior as revealed by an increased kurtosis of the generalization gradient, and a decreased width of model-derived generalization parameters. Moreover, D2R-blockade modulated similarity-based responses in the hippocampus and decreased midbrain-hippocampal connectivity, which in turn correlated with individual differences in generalization. These results suggest that dopaminergic activity in the hippocampus may relate to the degree of generalization and highlight a potential target for treatment.

**DOI:** http://dx.doi.org/10.7554/eLife.12678.001

## eLife digest

In the 1920s, two psychologists taught a young child known as ‘Little Albert’ to fear a white rat. They did so by striking a metal bar with a hammer whenever the rat was present. After experiencing the rat and noise together on multiple occasions, Little Albert eventually began to cry whenever the rat appeared. However, he also showed a similar response to a number of other white furry objects, including a rabbit and even a fur coat. By applying knowledge about a familiar object to other similar stimuli, humans and other animals avoid having to learn about each and every stimulus from scratch. However, this stimulus generalization is only effective if it occurs to the correct degree: under- or over-generalization (as shown by Little Albert) can lead to behaviors that are less than optimal.

Nerve cells use molecules called neurotransmitters to communicate with each other. For example, one nerve cell might release a neurotransmitter called dopamine, which can be detected on the surface of another nerve cell by a protein called the dopamine D2 receptor. Research suggests that dopamine is involved in stimulus generalization in rat and pigeons, but the effects of dopamine have not been studied in humans. Kahnt and Tobler have now explored how the degree of stimulus generalization is determined in human adults.

For the experiments, volunteers viewed images that contained sets of parallel lines, while lying inside a “functional MRI” brain scanner. The volunteers learned to associate lines with a particular orientation (e.g. 39° from vertical) with receiving a reward, and lines with another specific orientation with the absence of a reward. The next day, half the volunteers were given a drug that blocks the dopamine D2 receptor, while the other half received a placebo. All volunteers were then asked to classify lines of different orientations as either “rewarded” or “non-rewarded”.

Both groups classified lines that were aligned similarly to the previously rewarded orientation as also rewarded. However, those given the D2 receptor blocker classified a narrower range of line orientations as rewarded than those who received the placebo. In other words, blocking the D2 receptors reduced stimulus generalization. It also reduced activity in a region of the brain called the hippocampus, and the extent to which this activity was connected to activity in another area called the midbrain.

Taken together, these results suggest that activation of dopamine D2 receptors in the hippocampus may determine the extent to which we generalize between stimuli. Given that over- and under-generalization is a feature of psychiatric disorders such as depression and anxiety, manipulating D2 receptor activity could have therapeutic benefits in these patients.

**DOI:** http://dx.doi.org/10.7554/eLife.12678.002

## Introduction

Generalization enables neural systems to apply stimulus-outcome associations that have been acquired for one particular stimulus to other, related stimuli. A key aspect of generalization is the degree to which learned associations are applied to novel stimuli: the width of generalization. Generalization relieves individuals from having to learn outcome predictions for every single stimulus from scratch before using them to guide behavior. However, generalizing too widely can be maladaptive because it leads to the indiscriminate approach (or avoidance) of stimuli that are unlikely to be associated with reward (or punishment). Indeed, aberrant generalization is implicated in several neuropsychiatric diseases including schizophrenia, anxiety disorders, depression, and drug abuse (Dunsmoor and Paz, 2015; Gotlib and Joormann, 2010; Lissek et al., 2014b; Lucantonio et al., 2015; Moustafa et al., 2010; Shohamy et al., 2010). Insights into the neurobiological mechanisms controlling the width of generalization are therefore important for understanding adaptive behavior and its disruption in these conditions.

Two basic forms of generalization can be distinguished based on what constitutes the relation among stimuli; *associative* and *stimulus* generalization. In the case of associative generalization, such as transitive inference and acquired equivalence, the associative relationship among stimuli determines similarity. This relationship can be established for example by sensory preconditioning (e.g., train stimuli A-B and B-C, test whether A comes to predict C) or by a common associate (e.g. train A-C and B-C, test whether A and B are associated). In contrast, in stimulus generalization the relationship among stimuli is based on the similarity along one or more perceptual dimensions (frequency of sounds, color, line orientation, etc.). Most of what we know about stimulus generalization comes from behavioral experiments utilizing intradimensional stimulus discrimination (Dunsmoor and LaBar, 2013; Ghirlanda and Enquist, 2003; Guttman and Kalish, 1956; Hanson, 1959). In these paradigms, one stimulus (e.g. one particular line orientation) is paired with reward (rewarded conditioned stimulus, CS+), while a second stimulus (e.g. a slightly different line orientation), which differs from the first in only one dimension, is paired with no reward (unrewarded conditioned stimulus, CS−). Generalization is then tested using a range of stimuli that vary along the defining stimulus dimension (e.g. line orientation). Although the test stimuli have never been paired with reward, animals and humans show robust generalization in that they respond to test stimuli that are similar to the CS+. Several psychological models have been developed based on these experiments (Ghirlanda and Enquist, 1998; Pearce, 1987; 1994; Shepard, 1987; McLaren et al., 2012), but the neurobiological mechanisms regulating the width of stimulus generalization have remained unknown.

Early research suggested a role for dopamine in mediating generalization by demonstrating that blockade of dopamine receptors during generalization tests alters response gradients in rats and pigeons (Lyons et al., 1973a; 1973b; Terrace, 1963). While the effects of dopamine have not been investigated in humans, evidence from neuroimaging suggests that dopaminoceptive and dopaminergic regions such as the striatum and the midbrain are involved in generalization (Kahnt et al., 2012; Wimmer et al., 2012; Wimmer and Shohamy, 2012). However, because standard neuroimaging relies on indirect measurements of neural responses, these studies were unable to inform questions about neurotransmitter-specific activity.

A candidate brain region for a prominent role in stimulus generalization is the hippocampus, based on its involvement in learning, memory, and associative generalization (Dickerson and Delgado, 2015; Eichenbaum, 2000; Frank et al., 2006; Kumaran, 2012; Norman and O'Reilly, 2003; Squire and Wixted, 2011). Specifically, the hippocampus is thought to contribute to associative generalization by representing higher-order relationships among different stimuli (Eichenbaum, 1999; Howard et al., 2005; Kumaran and McClelland, 2012). However, human imaging and animal lesion studies suggest that the hippocampus may also be involved in similarity-based stimulus generalization not requiring inference (Casasola et al., 2007; Kahnt et al., 2012; Lissek et al., 2014a; Solomon and Moore, 1975).

In the current study, we directly examined the role of dopamine and hippocampal processing in regulating the width of stimulus generalization. For this purpose, we combined a visual intradimensional discrimination task (Kahnt et al., 2012) with pharmacologic dopamine D2-receptor (D2R) blockade (Kahnt et al., 2015) and functional magnetic resonance imaging (fMRI). Our central hypothesis was that dopamine is involved in regulating the width of stimulus generalization via the modulation of similarity-based processing in the hippocampus. In order to test this hypothesis, we fit a computational model of stimulus generalization to the behavioral data, and examined the model parameters regulating the width of generalization. We then used model-based fMRI and functional connectivity analyses to identify the brain processes that potentially mediate the effects of dopamine on generalization. We predicted that D2R blockade during the test session would reduce the computational parameters governing the width of generalization, and thus lead to narrower generalization gradients. Moreover, we hypothesized that this reduction would be mirrored in hippocampal activity as well as in reduced functional coupling between the dopaminergic midbrain and the hippocampus.

## Results

**Figure 1.**
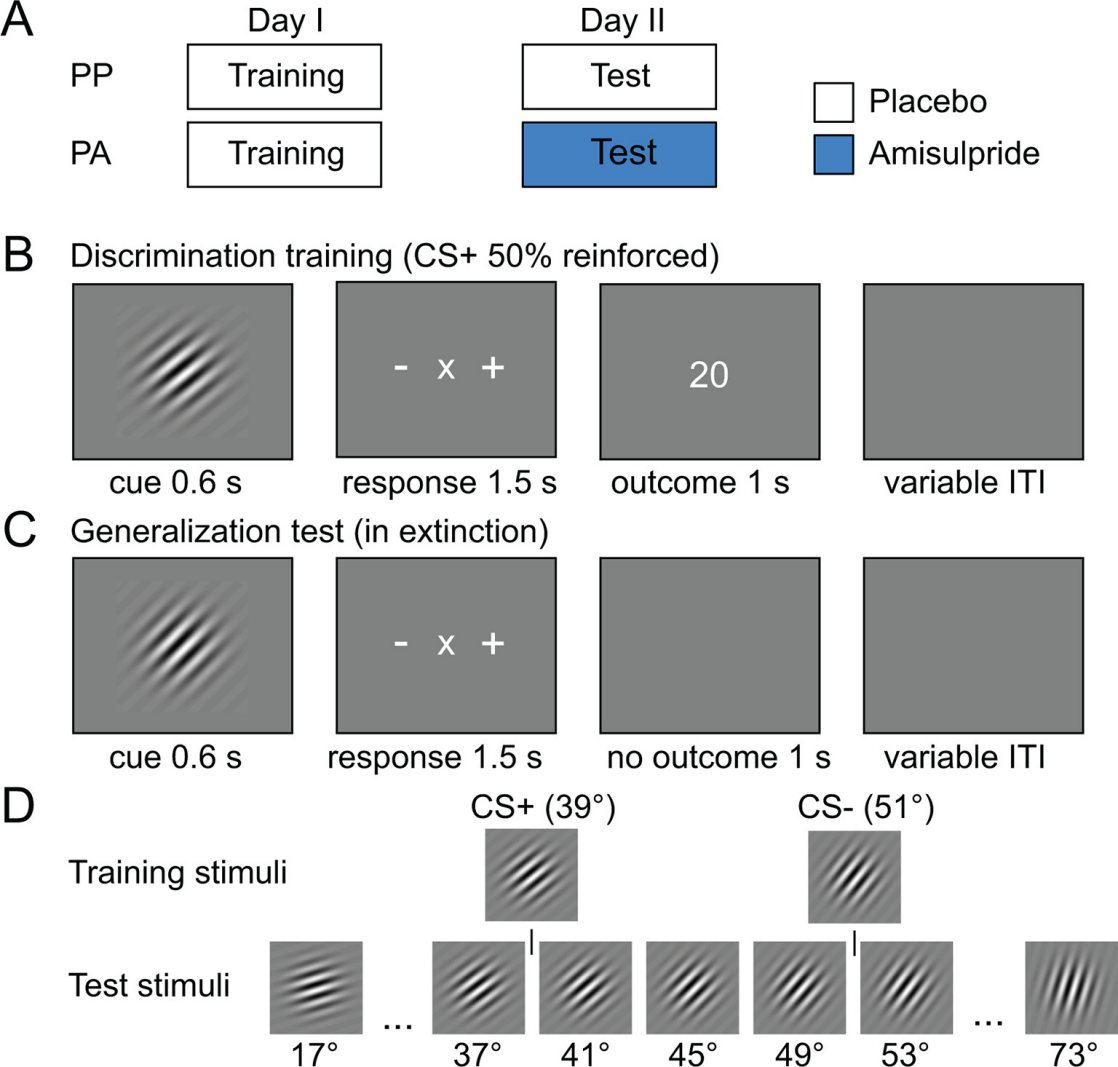
Experimental design and stimuli. (**A**) Subjects were pseudo-randomly assigned into a placebo-placebo (PP) or placebo-amisulpride (PA) group. Subjects in the PP group received placebo on both days, whereas subjects in the PA group received placebo before the discrimination training and amisulpride before the generalization test. (**B**) Discrimination training on day I. During each trial, one orientation (CS+ or CS–) was shown for 600 ms. Subjects had to indicate whether the current orientation may be rewarded (+) or not rewarded (–) using a button press. Outcomes were delivered independently of subjects’ response. After the response, the outcome was presented (20 or 0 cents). (**C**) Generalization test on day II. On each trial, subjects were presented with one of 15 different test orientations and indicated whether the current orientation was the one previously associated with reward (+), no reward (–) or neither (×). The mapping between buttons and +/–/× was randomized on each trial and the generalization test was performed in extinction. (**D**) Stimuli used during the training and test session. Associations between stimuli and outcomes were counterbalanced across subjects. **DOI:** http://dx.doi.org/10.7554/eLife.12678.003

The experiment was carried out on two consecutive days. On the first day, subjects underwent intradimensional discrimination training to learn stimulus-specific reward associations. On the second day, a generalization test was performed in extinction. All subjects received placebo on the first day, whereas on day two, one group received placebo (PP group) and the other group received 400 mg of the D2R blocker amisulpride (PA group) (Figure 1A). By verifying that the two groups learned equally well on day one, we ensured that any observed group differences in behavioral or neural responses during day two resulted from effects of dopamine on generalization at retrieval, independent of encoding-related effects. In the following sections we first describe the behavioral data during the training, and then examine how D2R blockade affected the neurocomputational mechanisms governing generalization using fMRI and a model of stimulus generalization.

### Robust acquisition of stimulus-outcome associations during discrimination training

**Figure 2.**
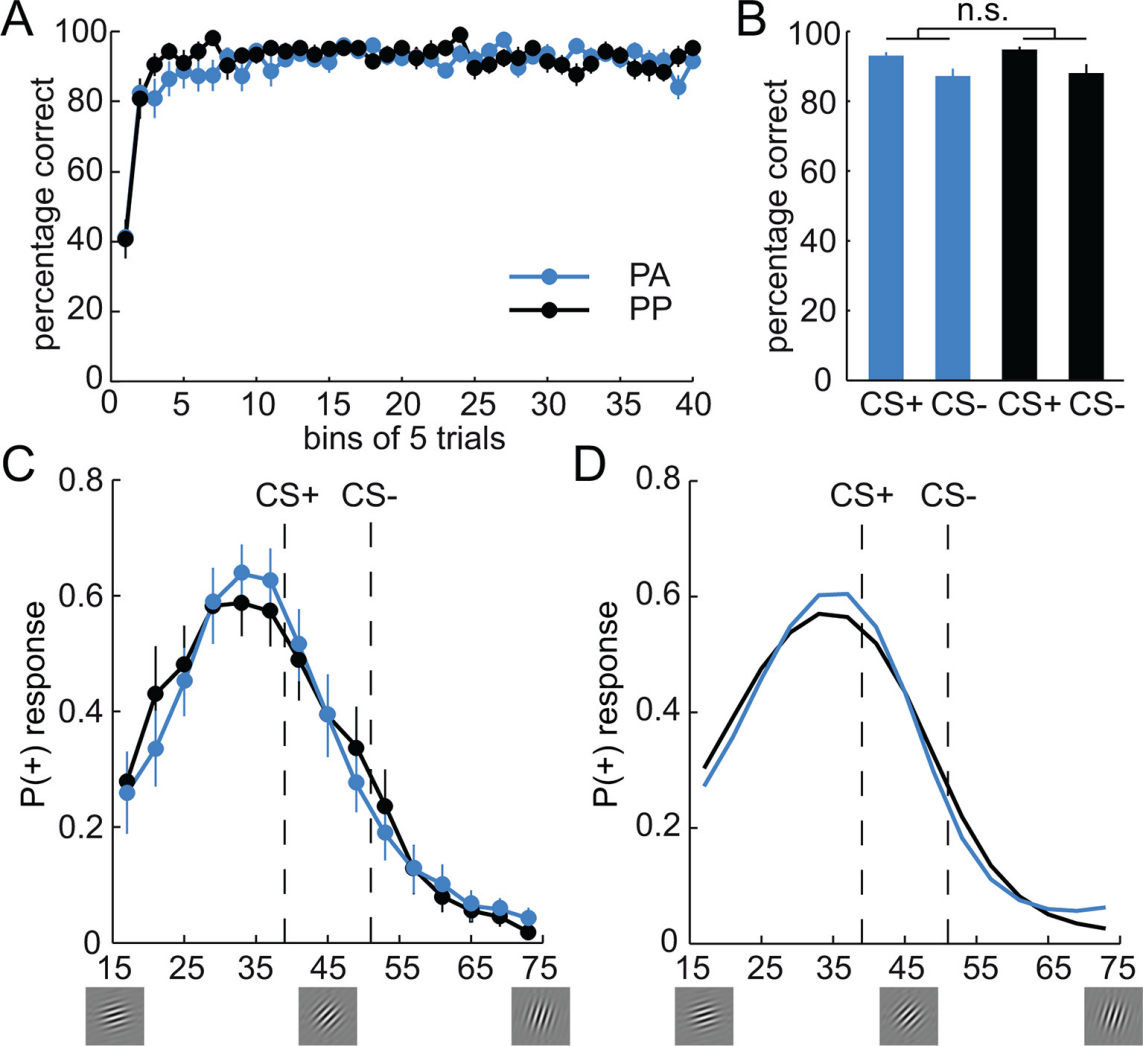
Behavioral responses during discrimination training and generalization test. (**A**) Percentage of correct responses during discrimination training across time (bins of 5 trials). Subjects learned the stimulus-outcome associations within the first 50 trials, and maintained performance at a high level afterwards. Given that both groups received placebo during the training session, performance was not expected to, and indeed did not, differ. (**B**) Percentage of correct responses for CS+ and CS− is plotted for both groups separately. (**C**) Generalization gradients reflect the probability of a + response as a function of stimulus orientation during the test session. Responses reveal a peak-shift (stronger responding on the side of the CS+ that is opposite to the CS−). Subjects in the amisulpride group (PA, blue), showed a narrower generalization gradient with a higher peak compared to subjects receiving placebo (PP, black). (**D**) Generalization gradients of a similarity-based generalization model with parameters estimated from subjects’ behavioral responses in both groups, separately. The model accurately reproduces the empirical generalization gradients and the differences between the groups. Error bars are SEM for N=25 (PA) and N=21 (PP). **DOI:** http://dx.doi.org/10.7554/eLife.12678.004

**Figure 3.**
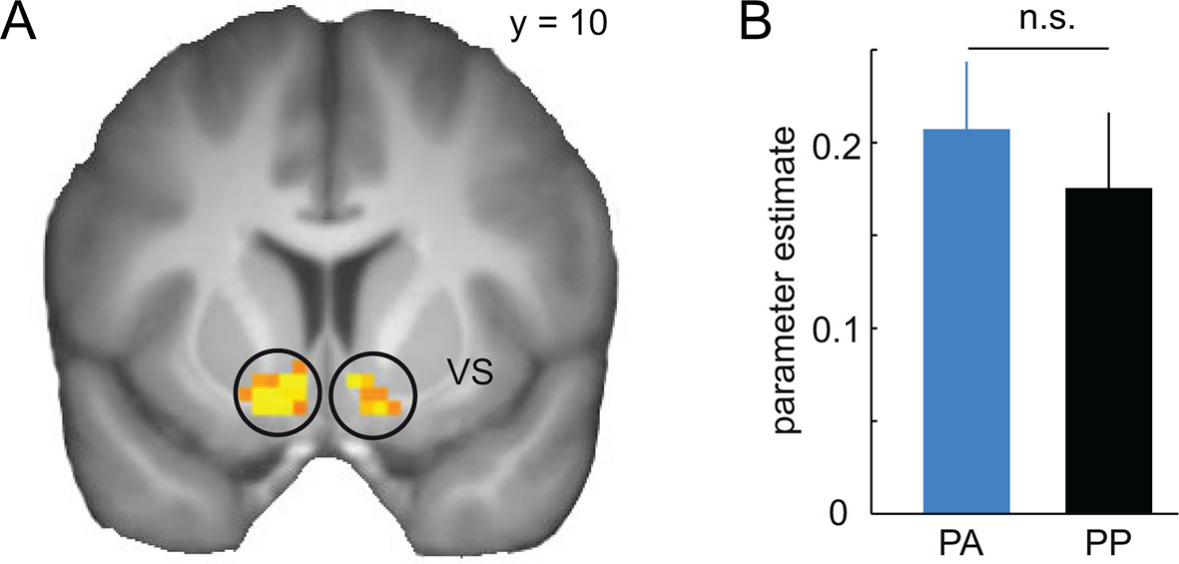
Prediction error responses in the ventral striatum during discrimination training. (**A**) Regions in the ventral striatum (VS, left, x = -15, y = 8, z = -16, t = 6.97, P < 0.001, FWE whole brain corrected; right, x = 6, y = 14, z = -10, t = 6.22, P = 0.003, FWE whole brain corrected) in which activity is correlated with model-derived prediction errors (PE) during the training session across both groups. T-map from one-sample t-test (across the two groups) is thresholded at P < 0.05, FWE whole brain corrected and overlaid on a T1-weigthed image averaged across subjects. (**B**) Bar plots depict parameter estimates for PE-related activity in the VS. Given that both groups received placebo during the training session, neural PE-responses were not expected to differ, and indeed did not differ between groups (two-sample t-test, t = 0.58, P = 0.56). Error bars are SEM for N = 25 (PA) and N = 21 (PP). **DOI:** http://dx.doi.org/10.7554/eLife.12678.006

The visual intradimensional discrimination task used the orientation of a Gabor patch as reward-relevant dimension (Figure 1B). Specifically, one orientation (39°) served as the CS+ and was paired with the delivery of 20 cents in 50% of trials, whereas a second orientation (51°) served as the CS− and was paired with no reward in all trials (Figure 1D). The association between orientation and reward was counterbalanced across subjects. In order to track the acquisition of stimulus-outcome associations, subjects were asked to make a discriminatory response after the stimulus was displayed, but before the outcome was shown. Subjects had to indicate whether the current stimulus was the rewarded stimulus, the non-rewarded stimulus, or whether they were unsure, by pressing buttons associated with +, -, or x, respectively. Subjects learned the discrimination within the first 50 trials and performed at high levels afterwards (Figure 2A). Performance increased as a function of time (two-way, group-by-time ANOVA, main effect of time, F(39,1716) = 18.98, P < 0.001), but did not differ between groups (main effect of group, F(1,44) = 0.41, P = 0.53; group-by-time interaction, F(39,1716) = 1.27, P = 0.13; two-way, group-by-CS type ANOVA, main effect of group, F(1,44) = 0.35, P = 0.56; group-by-CS type interaction, F(1,44) = 0.39, P = 0.53, Figure 2B). Moreover, learning-related activity in the ventral striatum (Delgado, 2007) did not differ between groups (see Figure 3), suggesting that both groups were also comparable in terms of neural responses during discrimination training. This demonstrates that, as expected, both groups acquired stimulus-outcome associations and performed at comparable levels during training. Accordingly, effects of D2R blockade during the generalization test session on the next day can be compared independent of potential group differences in discrimination training.

**Figure 2—figure supplement 1.**
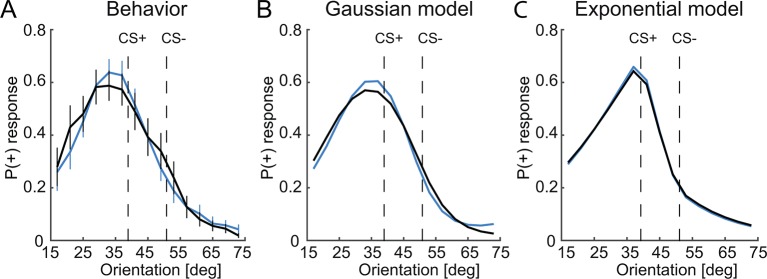
Comparison of Gaussian and exponential similarity functions. (**A**) Behavioral responses, (**B**) P(+) responses derived from models with a Gaussian and (**C**) exponential similarity functions. The model with the Gaussian similarity function better captures the behavior and the effect of the dopaminergic manipulation than the model with the exponential similarity function. **DOI:** http://dx.doi.org/10.7554/eLife.12678.005

### D2R blockade during test narrows generalization gradients

On the second day, subjects in the PP group received placebo, whereas subjects in the PA group received 400 mg of the D2R antagonist amisulpride (Rosenzweig et al., 2002). One hour later, subjects performed a generalization test session in extinction (without feedback). Specifically, on each trial, one of 15 orientations (17°–73°, Figure 1D) was presented, and subjects performed the same discrimination as during training (Figure 1C; the original CS+ and CS− orientations were not shown during the test). Responses described a bell-shaped gradient around the CS+ and revealed a peak shift (Derenne, 2010; Purtle, 1973; Wisniewski et al., 2009), such that subjects responded most frequently to an orientation that was never paired with reward (Figure 2C). Specifically, average responding was stronger to orientations on the side of the CS+ that was farther away from the CS− (paired t-test on responding to stimuli left vs. right of the CS+, t = 2.99, P = 0.006). Such peak shifts are typical for intradimensional discrimination with one CS+ and one CS−; they are thought to result from the summation of excitatory and inhibitory gradients around the CS+ and CS−, respectively, and have been observed across many species and stimuli (Ghirlanda and Enquist, 2003; Pearce et al., 2008; Spence, 1937).

Direct group comparisons of the individual data points along the generalization gradient did not reveal any significant differences (two-sample t-tests, all Ps > 0.29). However, visual inspection of the gradients suggested that the amisulpride group had a narrower gradient than the placebo group, with enhanced responding at the peak of the curve, reduced responding at both flanks, and enhanced responding at the tail of the curve. These shape features are parsimoniously described by the 4th moment of probability distributions, namely, their kurtosis. Accordingly, a test for differences in the kurtosis of group-specific distributions (Pearson type VII distribution, see Materials and methods) revealed a significantly greater kurtosis in the amisulpride group compared to placebo (PA: 6.73, PP: 3.29; permutation test, P = 0.043). This finding demonstrates that amisulpride narrowed the width and increased the peak of the behavioral generalization gradient, and suggests that D2R activity alters the neurocomputational processes that mechanistically control generalization behavior.

**Figure 4.**
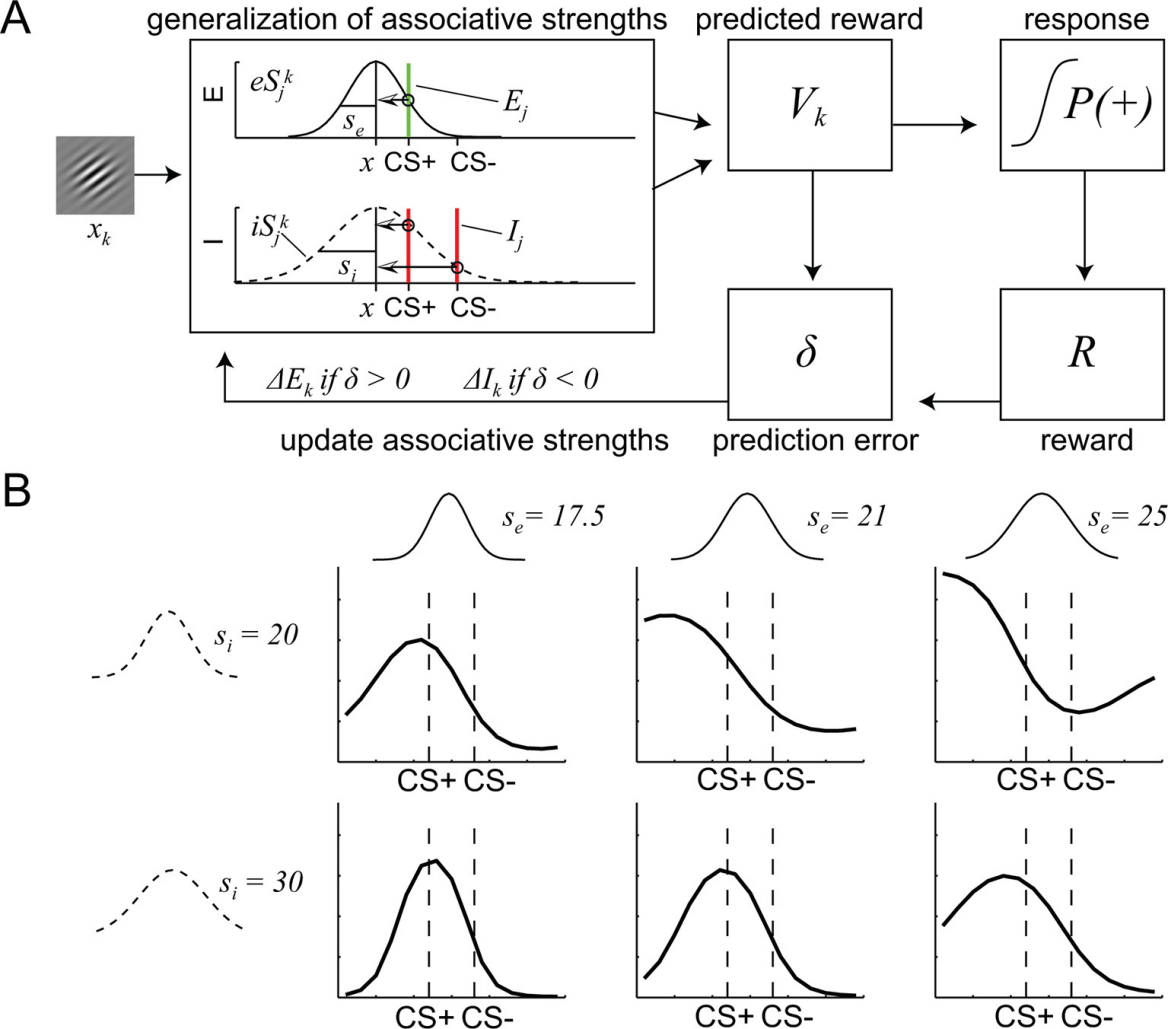
A computational model of similarity-based generalization. (**A**) Schematic of the model. When a stimulus orientation *x_k_* is presented, the predicted reward for this orientation *V_k_* is computed by integrating the excitatory and inhibitory associations *E* and *I* of all stimuli *j* that are similar to *k*, (including its own associations, *j* = *k*), weighted by the similarity between stimuli *j* and *k*. The similarity between *j* and *k* is determined by the excitatory and inhibitory generalization coefficients *eS_j_^k^* and *iS_j_^k^*, respectively, which are assumed to be Gaussian (or exponential, not shown here). The width of the excitatory and inhibitory generalization coefficients, and thus the degree to which excitatory and inhibitory associations generalize from *j* to *k* is determined by the parameters *s_e_* and *s_i_*. The reward prediction *V_k_* is used to generate approach behavior *P(+)* and to compute a reward prediction error *δ*, which in turn updates the excitatory and inhibitory associations of *k*. (**B**) Illustration of the effects of changes in the width of excitatory and inhibitory generalization coefficients on generalization gradients. **DOI:** http://dx.doi.org/10.7554/eLife.12678.007

To further address this possibility, and to identify the specific computational parameters that are affected by D2R blockade, we utilized a mathematical model for similarity-based stimulus generalization (see Materials and methods and Figure 4). The model assumes that the reward prediction of a given stimulus reflects the integrated excitatory and inhibitory associations of that stimulus, plus the excitatory and inhibitory associations of stimuli that are similar to it (Pearce, 1987). Critically, associations of stimuli that are similar to the currently presented stimulus have a stronger contribution than the associations of dissimilar stimuli. Because the shape of the function determining the similarity between the currently presented stimulus and other stimuli (i.e. the generalization coefficient) is of critical importance (Ghirlanda and Enquist, 2003), we directly compared the most commonly used models, i.e. one with Gaussian (Kahnt et al., 2012) and the other with exponential similarity functions (Shepard, 1987). While the exact shape of the similarity functions differs between models, for both models, the extent to which inhibitory and excitatory associations generalize to the current stimulus is controlled by the width of the similarity functions (*s_i _*and *s_e_*) (Figure 4B). The larger *s_i _*and *s_e_*, the stronger the impact of the inhibitory and excitatory associations of dissimilar stimuli on the currently predicted reward, respectively, and thus the more generalization takes place.

In order to assess the explanatory power of the Gaussian and exponential similarity functions, we directly compared their fit to the behavioral data. The free parameters of both models (width of inhibitory generalization coefficient, *s_i_*; width of excitatory generalization coefficient, *s_e_*; slope, *β*; offset, *a*; learning rate, *α*) were estimated for the entire group of subjects by maximizing the likelihood of subjects’ responses during the generalization test given the model (see Materials and methods). Visual inspections suggested that the model with the Gaussian similarity function fitted the behavioral data better than the model with the exponential similarity function (Figure 2—figure supplement 1). This was confirmed by a formal model comparison using the Akaike information criterion (AIC) and Bayesian information criterion (BIC) (Gaussian: AIC = 9586.4, BIC = 9629.2; Exponential: AIC = 9691.6, BIC = 9734.4). We also compared the fit of the two models by comparing the regression coefficients from a logistic regression of the trial-by-trial responses on the modeled P(+) responses. Although both models predicted behavioral responses reliably (Gaussian: t = 11.06, P < 0.001; exponential: t = 11.38, P < 0.001), we found significantly higher regression coefficients for the Gaussian model (paired t-test t = 5.34, P < 0.001). Taken together, this demonstrates that in our experiment, a Gaussian similarity function fits behavior better than an exponential similarity function.

**Figure 5.**
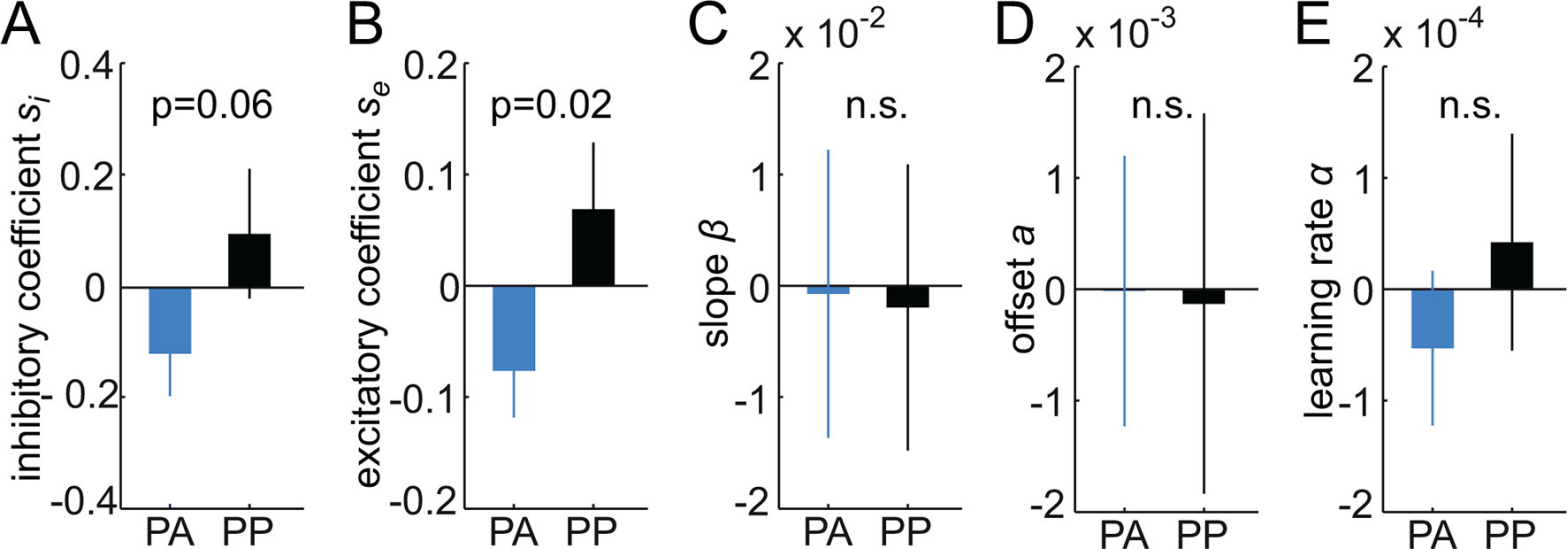
Estimates of individual differences in model parameters. (**A-–E**) Each bar plot depicts the average difference between model parameters estimated based on the whole group (N) and a reduced group when one subject is left out (N-1). Values are averaged according to the group of the left out subject (i.e. PA or PP). Positive values indicate that removing subjects from the group decreased the parameter estimates in the N-1 group. Amisulpride reduced parameters controlling the width of generalization (width of inhibitory and excitatory generalization coefficients, A, B) but had no effect on the slope (**C**) and offset of responding (**D**), as well as the learning rate (**E**). P-values are based on two-sample t-tests. Error bars are SEM for N = 25 (PA) and N = 21 (PP). **DOI:** http://dx.doi.org/10.7554/eLife.12678.008

To determine the effects of dopamine on generalization, in a next step, the free parameters of the Gaussian model were estimated separately for each group (see Materials and methods). As can be seen in Figure 2D, the model (with group-specific parameters) accurately reproduced responses in both groups, including the differences in the shape of the generalization gradients. Logistic regression coefficients were significantly different from zero (PA: t = 7.76, P < 0.001; PP: t = 7.95, P < 0.001), and did not differ between groups (t = -0.21, P = 0.84), suggesting that responses in both groups were well described by the model. Notably, comparing the model parameters between groups (Table 1), revealed significant group differences in the width of the excitatory generalization coefficient, with a smaller coefficient in the amisulpride group compared to the placebo group (permutation test, P = 0.035). The width of the inhibitory coefficient was also smaller in the amisulpride group, but this difference was not significant (P = 0.069). Importantly, the learning rate during test did not differ between groups (P = 0.21), demonstrating that additional learning during the test session, which might have been altered by amisulpride, cannot account for the differences in generalization gradients. In order to obtain individual estimates of model parameters, we re-estimated the model using a leave-one-out procedure (see Materials and methods). Comparing the resulting individual estimates between groups confirmed a significant difference in the width of the excitatory (two-sample t-test, t = -2.03, P = 0.024), and to a lesser degree, the inhibitory generalization coefficient (t = -1.59, P = 0.059, Figure 5). These results suggest that D2R blockade modulates the computational processes that control the width of stimulus generalization, resulting in narrower generalization.

**Table 1.** Group-specific model parameters. **DOI:** http://dx.doi.org/10.7554/eLife.12678.009

Group	Inhibitory coefficient s_i_	Excitatory coefficient s_e_	Slope β	Offset a	Learning rate α_test_
PA group N=25	20.121	17.599	3.093	0.370	0.002
PP group N=21	30.587	24.584	3.124	0.361	0.006
Difference PA-PP	-10.465	-6.985	-0.031	0.009	-0.004
*P-value (PA-PP)	0.069	0.035	0.495	0.462	0.214

Note: *P-value is based on 10.000 permutations.

**Figure 6.**
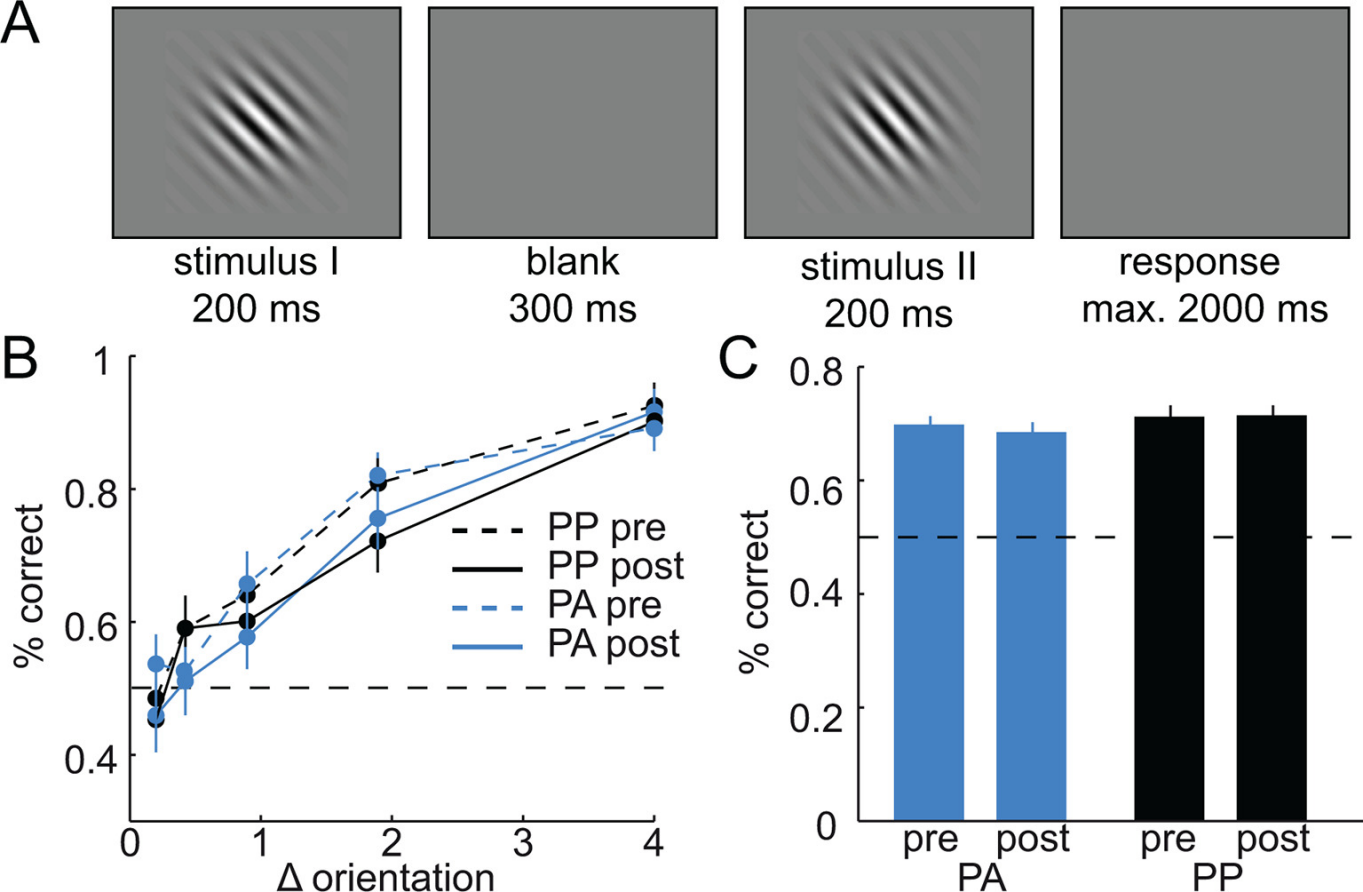
Amisulpride does not enhance perceptual discrimination performance. (**A**) Design and timing of the perceptual orientation discrimination task. Stimulus I was always 135°, whereas stimulus II was tilted by ± 0.2°, ± 0.4°, ± 0.9°, ± 1.9°, or ± 4°. Subjects had to indicate whether stimulus II is tilted counter-clockwise or clockwise relative to stimulus I. (**B**) Performance differed as a function of the difference in orientation between stimulus I and stimulus II (3-way ANOVA, F(4,172) = 74.46, P < 0.001), but not between groups (F(1,43) = 0.01, P = 0.93) or time (pre vs. post drug, F(1,43) = 2.73, P = 0.11). Importantly, there was no significant group-by-time interaction (F(1,43) = 0.07, P = 0.80). (**C**) Same data as in B, but collapsed across levels of orientation difference. Again, performance did not differ between groups (two-way ANOVA, F(1,43) = 0.99, P = 0.33), time (F(1,43) = 0.22, P = 0.65), and there was no significant group-by-time interaction (F(1,43) = 0.42, P = 0.52). Error bars are SEM for N = 24 (PA) and N = 21 (PP). **DOI:** http://dx.doi.org/10.7554/eLife.12678.010

In principle, the observed effects of amisulpride on the width of generalization could have resulted from a drug-induced improvement in perceptual orientation-discrimination. To control for such perceptual effects, subjects performed a challenging orientation discrimination task, once before the drug took effect and once after (see Materials and methods and Figure 6). Performance (percentage correct) on this task did not differ between groups (two-way, time-by-group ANOVA, main effect of group, F(1,43) = 0.99, P = 0.33) and there was no group-by-time interaction (F(1,43) = 0.42, P = 0.52). Moreover, while perceptual discrimination performance was reliable across time (correlation between pre- and post-drug performance, r = 0.52, P < 0.001), it was not related to the width of the estimated generalization coefficients (all Ps > 0.38). Taken together, this control analysis demonstrates that the effects of amisulpride on generalization cannot be explained by perceptual improvements in orientation discrimination per se.

### D2R blockade reduces similarity-based activity in the hippocampus

**Figure 7.**
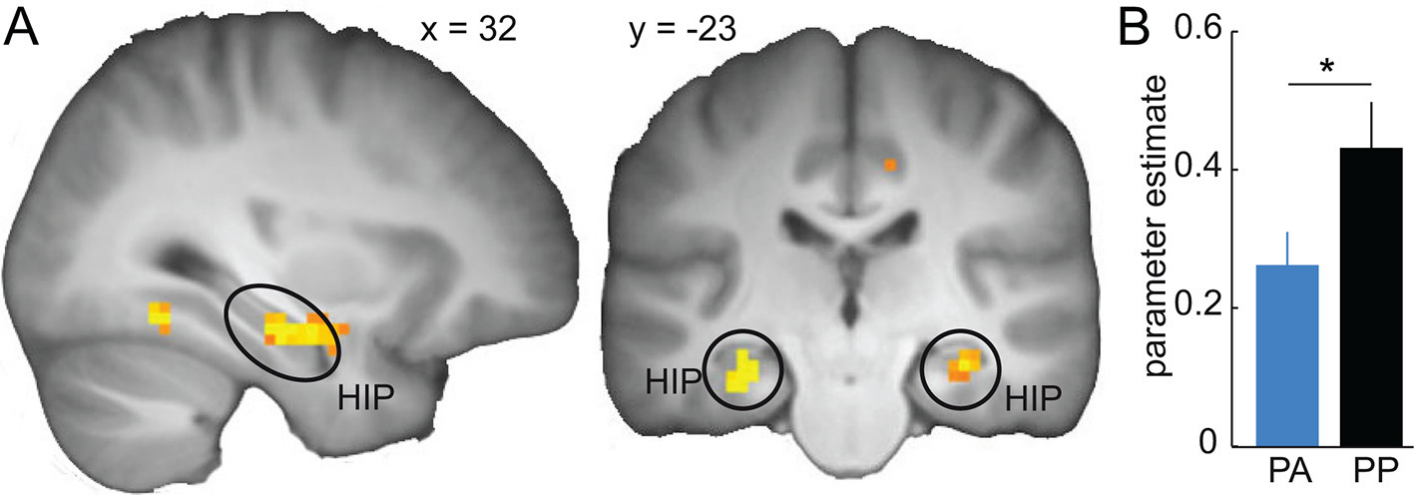
Similarity-based prediction error responses in the hippocampus during generalization. (**A**) Across both groups, activity in the hippocampus is significantly correlated with model-derived prediction errors during generalization test. T-map from one-sample t-test is thresholded at P < 0.05, FWE whole brain corrected and overlaid on a T1-weigthed image averaged across subjects. (**B**) Prediction error-related activity in the hippocampus is significantly reduced by D2R blockade (two-sample t-test, t = -2.12, P = 0.02). Error bars are SEM for N = 25 (PA) and N = 21 (PP). **DOI:** http://dx.doi.org/10.7554/eLife.12678.011

Having established an effect of D2R blockade on the computational processes that govern stimulus generalization, we next examined the neural circuits that mediate these changes. We first identified brain regions involved in generalization of reward predictions during retrieval. As a proxy of generalized value, we focused on prediction error responses derived from our model, which reflect the extent to which reward predictions have generalized from the original CS+ and CS− to the current stimulus (please note that because no outcomes were shown, prediction errors are perfectly but negatively correlated with expected value). Accordingly, to identify brain regions involved in similarity-based computations during generalization, we searched for regions in which fMRI activity correlated with generalized prediction errors at the time of the expected outcome. Based on previous empirical and modeling work linking hippocampal activity to the representation of relationships between stimuli and their predicted value (Kumaran et al., 2009; Kumaran and McClelland, 2012; Lee et al., 2012; Lissek et al., 2014a; Wimmer and Shohamy, 2012), we expected fMRI signals in the hippocampus to positively correlate with generalized prediction errors. In line with this hypothesis, across the entire group (one sample t-test) we found significant correlations in the bilateral hippocampus (extending into the parahippocampal gyrus, left, x = -30, y = -22, z = -16, t = 6.53, P = 0.001, FWE whole brain corrected; right, x = 33, y = -19, z = -16, t = 7.11, P < 0.001, FWE whole brain corrected, Figure 7A). Similar effects were found in the left amygdala (x = -24, y = -4, z = -19, t = 6.04, P = 0.006, FWE whole brain corrected) and the bilateral middle temporal gyrus (left, x = -48, y = -73, z = 14, t = 6.41, P = 0.002, FWE whole brain corrected; right, x = 28, y = -64, z = 14, t = 5.57, P = 0.022, FWE whole brain corrected). In addition, supporting recent work highlighting a role for medial (mPFC) and ventromedial PFC (vmPFC) in generalization (Dunsmoor and Paz, 2015; Onat and Buchel, 2015), at an uncorrected threshold of P < 0.001, we found a cluster in the mPFC (x = -3, y = 56, z = 8, t = 4.03) extending into the vmPFC.

We next tested whether reduced behavioral generalization observed in the amisulpride group was paralleled by a decrease in generalization-related activity in the hippocampus. In line with this idea, we found significantly reduced activity in the hippocampus in the amisulpride relative to the placebo group (two-sample t-test, t = -2.12, P = 0.02, Figure 7B). To examine whether these effects of dopamine on generalization-related activity are specific to the hippocampus, as a control, we tested for similar group differences in the amygdala, middle temporal cortex and mPFC. No significant group differences were observed in the amygdala (P = 0.43), the middle temporal gyrus (P = 0.31), or the medial PFC (P = 0.98). However, post-hoc analyses directly comparing the effect of the drug in the hippocampus to the drug effect in the other regions (i.e. group-by-region interactions), demonstrated that while the effect of D2R blockade in the hippocampus was significantly stronger than in the mPFC (P = 0.02) similar interactions involving the amygdala (P = 0.097) and the middle temporal gyrus (P = 0.127) did not reach significance. These data suggest specificity of the effects of D2R blockade on similarity-based processing in the hippocampus relative to the mPFC, but not necessarily relative to the amygdala and middle temporal lobe.

### Midbrain-hippocampal connectivity correlates with the width of generalization

**Figure 8.**
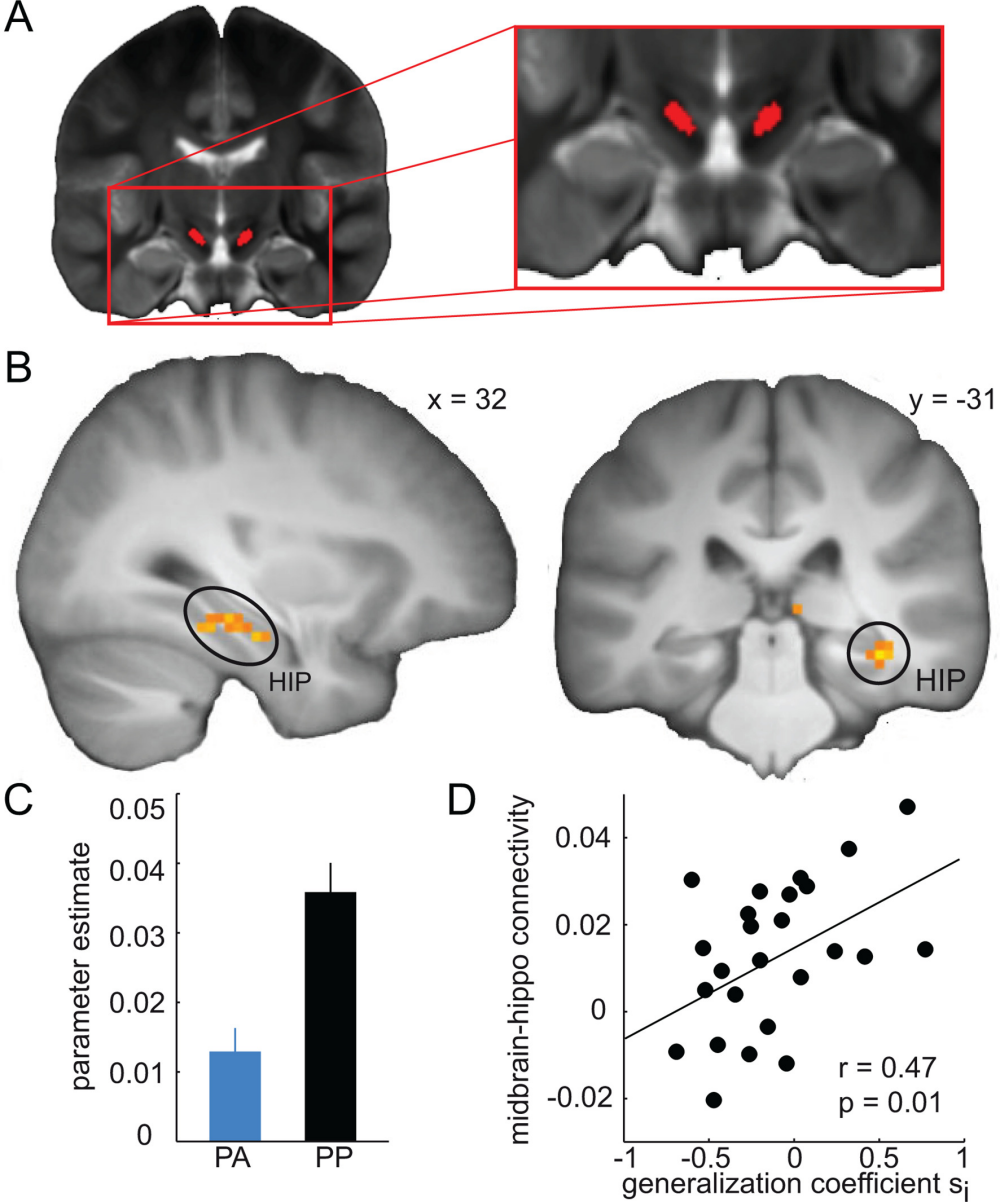
Amisulpride modulates midbrain-hippocampal connectivity. (**A**) Anatomical seed region in the midbrain is overlaid on a T2-weigthed image averaged across subjects. (**B**) Functional connectivity between the midbrain and the hippocampus during generalization test is significantly reduced in the amisulpride group (P < 0.05, FWE small volume corrected). For illustration, t-map from two-sample t-test (PP > PA) is thresholded at P < 0.001, uncorrected and overlaid on a T1-weigthed image averaged across subjects. (**C**) For illustration, bar plot depicting average midbrain-hippocampus connectivity in both groups. (**D**) Strength of midbrain-hippocampal connectivity is positively correlated with the width of the model-estimated inhibitory generalization coefficient (r = 0.47, P = 0.01) in the amisulpride group. Error bars are SEM for N = 25 (PA) and N = 21 (PP). **DOI:** http://dx.doi.org/10.7554/eLife.12678.012

In a next step, we examined the neural pathways on which DR2 blockade may mediate its effects on generalization. Given the anatomical origin of the dopaminergic projections to the hippocampus (Swanson, 1982), the relevance of hippocampal D2R for memory functions (Takahashi et al., 2008), and the modulation of hippocampal processing reported above, we hypothesized that amisulpride would reduce the functional connectivity between the midbrain and the hippocampus. In line with this prediction, a functional connectivity analysis with the midbrain as a seed region (Figure 8A, see Materials and methods) revealed decreased midbrain connectivity in the right hippocampus (x = 33, y = -19, z = -19, t = 3.68, P = 0.02, FWE small volume corrected, Figure 8B,C) and the left striatum (x = -9, y = 8, z = -19, t = 4.26, P = 0.001, FWE small volume corrected), in the amisulpride compared to the placebo group. This finding suggests that D2R blockade may modulate the functional connectivity between the midbrain and dopaminergic target regions such as the hippocampus and the striatum. To examine the specificity of these findings, we tested for similar drug-related effects on functional connectivity in the regions involved in similarity-based processing defined above. While connectivity estimates differed significantly between groups in middle temporal gyrus (P = 0.01), no drug effects were observed in the amygdala (P = 0.296) and the mPFC (P = 0.17). Accordingly, for all regions except the middle temporal gyrus (P = 0.13), the corresponding drug-by-region interactions were significant (all Ps < 0.05), suggesting that amisulpride-related decreases in midbrain connectivity are relatively specific to the hippocampus.

Finally, we examined whether the relationship between D2R blockade and the width of generalization is associated with midbrain-hippocampal connectivity. Specifically, we tested the correlation between midbrain-hippocampal connectivity and the estimated width of generalization coefficients in the amisulpride group. This correlation was significant for the inhibitory generalization coefficient (r = 0.47, P = 0.01, Figure 8D), but not for the excitatory coefficient (r = 0.18, P = 0.19). We replicated these findings in our previous data set (Kahnt et al., 2012), showing that midbrain-hippocampal connectivity was significantly correlated with the inhibitory generalization coefficient (r = 0.44, P = 0.036), but not with the excitatory coefficient (r = 0.15, P = 0.49). Interestingly, this relationship was not observed for midbrain-striatal connections (P > 0.16), and was significantly stronger for midbrain-hippocampal compared to midbrain-striatal connectivity (Z = 2.31, P = 0.011), suggesting that the relation between dopamine and the width of generalization may arise primarily from midbrain-hippocampal connections. In summary, these findings suggest a link between dopamine-mediated midbrain-hippocampal coupling and the width of stimulus generalization in humans.

## Discussion

The degree to which individuals generalize outcome predictions across similar stimuli is important for adaptive behavior. Here, using D2R pharmacology, fMRI, and computational modeling, we demonstrate that D2R blockade results in narrower behavioral generalization gradients and changes in the computational parameters controlling the width of generalization. Moreover, D2R blockade altered similarity-based processing in the hippocampus and decreased the functional coupling between the midbrain and the hippocampus. This coupling was in turn related to the computational parameter controlling the width of generalization.

Previous empirical and modeling work suggests that the hippocampus contributes to generalization by detecting the relationship between items in memory (Eichenbaum, 1999; Howard et al., 2005; Kumaran and McClelland, 2012). However, this function was thought to only apply to associative forms of generalization involving higher-order relationships, whereas basic stimulus generalization involving perceptual similarity is suggested to be hippocampus-independent (Kumaran, 2012). Opposing this view, here we provide evidence that the hippocampus is involved in stimulus generalization. Accordingly, our findings suggest that hippocampal similarity computations are not restricted to detecting higher-order relationships among stimuli as previously thought, but can also exploit the perceptual similarity between stimuli to establish meaningful relationships. While the hippocampus may not be necessary for stimulus generalization per se, our data suggest that it facilitates generalization by allowing a flexible modulation of its width. Specifically, such flexibility could not be achieved if discrimination and generalization were entirely based on static, hippocampus-independent, stimulus-outcome associations.

Our results contribute to an ongoing debate regarding the time point at which hippocampal processes support generalization. Two alternative accounts suggest that the relationship among stimuli is established by the hippocampus either during encoding via overlapping neural codes (Eichenbaum, 1999; Howard et al., 2005), or at the time of retrieval by means of recurrent similarity computations that are based on separated neural codes (Kumaran and McClelland, 2012). The former proposal has received empirical support from studies showing that hippocampal activity during encoding is related to transfer performance at test (Shohamy and Wagner, 2008; Wimmer and Shohamy, 2012). In contrast, in line with the second account, our model assumes that reward predictions are generalized and integrated during test (Pearce, 1987). It should be noted though that this model is mathematically equivalent to a model in which generalization occurs at encoding (Kahnt et al., 2012), and thus, the model alone does not provide evidence for either account. However, only if generalization occurs at retrieval can the width of generalization be modulated after encoding has occurred, and thus, our results are only compatible with a retrieval-based account of generalization. Specifically, because dopamine receptor blockade modulated generalization gradients during test, without affecting additional learning, our pharmacological manipulation provides evidence that stimulus generalization occurs – at least in part – at retrieval.

Our data suggest that the functional connectivity between the dopaminergic midbrain and the hippocampus is related to the width of generalization. Specifically, we found that participants with D2R blockade showed decreased midbrain-hippocampal connectivity, which in turn correlated with the width of generalization. While the presence of dopamine receptors in the hippocampus is undisputed, it is worth keeping in mind that although direct dopaminergic innervation from the midbrain to the hippocampus is present (Gasbarri et al., 1994) and functionally relevant for memory stabilization (McNamara et al., 2014), this pathway is not very strong (Mingote et al., 2015; Swanson, 1982). Moreover, it is conceivable that some of the hippocampal dopamine is co-released from noradrenergic neurons, in which it may not have been completely metabolized.

As such, our data is in line with the idea that reduced activity of hippocampal D2R decreases the extent to which reward associations generalize across stimuli. By extension, enhanced dopaminergic activity in the hippocampus may increase the width of generalization. More specifically, dopamine release in the hippocampus could increase the likelihood that ensemble patterns representing similar stimuli are activated, which would effectively lower the threshold for similarity detection, and, by broadening the range of stimuli for which reward predictions are taken into account, facilitate generalization. Conversely, reduced levels of dopamine would decrease the likelihood of ensemble pattern activation, increase the threshold for similarity detection, and, by enhancing fine-tuned discrimination, reduce generalization. This provides a simple neurobiological mechanism by which dopamine may flexibly adjust generalization during retrieval. Such flexibility is highly adaptive as it allows for different levels of generalization based on the state of the organism or the environment. We speculate that tonic dopamine levels, similar to their enabling effects on movements (Schultz, 2007), play an enabling role in generalization. For instance, in situations where dopamine transmission is high, such as in novel environments (Ihalainen et al., 1999; Lisman and Grace, 2005), elevated levels of dopamine may not only support memory formation (Shohamy and Adcock, 2010), but also broaden generalization and thereby facilitate exploratory behavior.

Of note, although D2R blockade reduced generalization coefficients for both excitatory and inhibitory associations (albeit less reliably), midbrain-hippocampal connectivity was only correlated with the width of the inhibitory coefficient. This raises the possibility that only the effects of D2R blockade on the inhibitory coefficient are mediated via a modulation of midbrain-hippocampal connectivity, whereas the effects on the excitatory coefficient are mediated via a different, yet to be explored, mechanism. The dissociation between inhibitory and excitatory generalization coefficients is in line with the interpretation of previous results suggesting that administration of chlorpromazine, a nonspecific dopamine receptor antagonist, specifically reduces the strength of inhibitory associations in a dose-dependent manner (Lyons et al., 1973b; Terrace, 1963). Moreover, it adds to previous findings indicating that generalization involving appetitive and aversive outcomes may involve different mechanisms (Schechtman et al., 2010).

By revealing the effects of dopamine on generalization, our current results substantially extend those of our previous study (Kahnt et al., 2012). Specifically, the current experiment suggests an association between dopamine and the width of generalization in the hippocampus, and dissociates effects of generalization during retrieval vs. encoding, which could not be achieved with the previous design. However, whereas here we find that prediction errors correlate primarily with activity in the hippocampus, the previous study identified prediction error related activity primarily in the ventral striatum. Also, in the previous study functional connectivity between the striatum and the hippocampus was related to the modeled generalization coefficients, whereas our current results suggest a facilitating role of midbrain-hippocampal connections. Several notable differences in the design of both experiments might explain these discrepancies. Most importantly, the previous design consisted of multiple alternating training and testing blocks, all conducted within one session on a single day, whereas in the current study, training and test did not alternate but were conducted in distinct sessions that were separated by a 24 hr delay. Moreover, in the 2012 study, CS-outcome associations were deterministic (100% contingency), whereas the current experiment involved a 50% reinforcement schedule in order to slow down extinction during test. These differences could have shifted the primary focus of generalization-related processing from the striatum to the hippocampus.

In conclusion, here we propose a neurobiological mechanism for the control of stimulus generalization, in which midbrain dopamine changes similarity computations in the hippocampus, resulting in altered generalization gradients. As such, our results demonstrate that the width of stimulus generalization is not hard-wired but flexible, and can change under pharmacological interventions. Accordingly, our results have important clinical implications for a number of neuropsychiatric disorders in which generalization is disrupted. Specifically, aberrant generalization is implicated in depression, anxiety, and schizophrenia (Buss and Daniell, 1967; Gotlib and Joormann, 2010; Lissek et al., 2014b; Shohamy et al., 2010), and our findings indicate that blocking D2R activity may provide a potential treatment of overgeneralization in these disorders.

## Materials and methods

### Subjects

Subjects were assigned to one of three groups in a double-blind and pseudo-random fashion: amisulpride-placebo (AP), placebo-amisulpride (PA), and placebo-placebo (PP). To avoid confounds related to the effects of dopamine on neural processing during discrimination training, only subjects in the PA and PP group are considered in this manuscript. A total of seven subjects was excluded because they either failed to acquire stimulus-outcome associations during the discrimination training on day one (performance <60%, 4 subjects) or because they failed to follow instructions on day two (3 subjects). Subjects in both groups received placebo on the first day of the experiment, whereas on the second day subjects in the PP and PA group received placebo and 400 mg of the D2R blocker amisulpride, respectively (Figure 1A). All subjects were healthy and had normal or corrected-to-normal vision. Groups did not differ significantly in number (PA: N = 25, PP: N=21, chi-square = 0.348, P = 0.56), average age, (PA: 22.72 years ± 2.17 SD, PP: 22.19 years ± 1.83 SD; t = 0.88, P = 0.38), and average weight (PA: 75.04 kg ± 7.89 SD, PP: 75.48 kg ± 10.92 SD; t = -0.03, P = 0.98). Moreover, subjects were not aware of whether they received placebo or amisulpride on both days as assessed by a post experimental questionnaire (day I, chi-square = 0.49, P = 0.48, day II: chi-square = 0.29, P = 0.60). The study was approved by the Cantonal Ethics Review Board of Zurich, and subjects provided informed consent to participate.

### Experimental design and stimuli

On the first day, subjects were briefed about the details of the experiment, signed the consent form, and were administered a pill that was swallowed in front of the experimenter. To minimize and equalize absorption time across subjects, subjects were asked to not eat 6 hr before the experiment.

One hour after taking the pill, subjects entered the MRI scanner to perform an intradimensional discrimination task. During the task, subjects learned the association between oriented Gabor patches (CS+ and CS−) and reward or no reward (0 and 20 cents, respectively). In each trial, subjects were presented with an oriented Gabor patch for 600 ms (Figure 1B). Immediately after the stimulus, subjects had to indicate whether the currently displayed stimulus may lead to reward (+) or no reward (-), or whether they did not know (x) by pressing a button with the index, middle, or ring finger of their right hand, corresponding to the signs (+/-/x) on a response mapping screen. The mapping between buttons (fingers) and +/-/x was randomized in each trial to dissociate signals related to motor preparation and execution from reward predictions and prediction errors. When subjects pressed a button, the brightness of the signs on the screen slightly decreased to indicate that a response has been made. The screen disappeared after 1500 ms (maximum decision time) and was replaced by an outcome screen (1000 ms) indicating the amount of money they received (20 or 0 cents). When subjects failed to respond within 1500 ms, “‘too slow”’ was presented instead of the outcome. The CS+ was paired with reward and no reward in 50% of the trials, whereas the CS− was always paired with no reward. The outcome was independent of the correctness of the behavioral response, and the association between stimulus orientation (39° and 51°) and reward was counterbalanced across subjects. The training phase consisted of 100 repetitions of CS+ and CS− trials, in pseudorandom order. Trials were separated by a variable interval ranging from 1.9 to 9.9 s (1.9 s fix, plus a variable interval drawn from an exponential distribution, truncated at 8 s).

On the second day of the experiment, subjects performed the generalization test in extinction, one hour after taking the pill containing either placebo (PP) or amisulpride (PA). In each trial, subjects saw one of 15 orientations (17°, 21°, 25°, 29°, 33°, 37°,41°, 45°, 49°, 53°, 57°, 61°, 65°, 69°, and 73°; Figure 1D) for 600 ms (Figure 1C). The original CS+ and CS− were not shown during the test. Each orientation was presented 14 times in pseudorandom order resulting in a total of 210 trials. Directly after presentation of the stimulus, subjects had to make the same discrimination response as during training (see above). Importantly, the test was performed in extinction, i.e. no outcomes were shown for all orientations. This design ensured that subjects made motor responses to all stimuli and thus allowed us to observe reward prediction error responses to all orientations independent of potential confounds attributable to reward feedback, different visual stimulation, and different cognitive or motor demands. Trials were separated by a variable interval ranging from 2.9 to 10.9 s (2.9 s fix, plus a variable interval drawn from an exponential distribution, truncated at 8 s).

### Orientation discrimination performance

In order to control for potential effects of D2R blockade on perceptual performance per se, subjects performed an orientation discrimination task. In each trial, two oriented Gabor patches were presented for 200 ms each, separated by a blank screen of 300 ms. The first stimulus had an orientation of 135° (i.e.orthogonal to the stimuli used in the main experiment), whereas the second stimulus was tilted -4°, -1.9°, -0.9°, -0.4°, -0.2°, 0.2°, 0.4°, 0.9°, 1.9°, or 4° relative to the first stimulus. Thus, the task consisted of 10 trial types, reflecting 5 levels of difficulty (i.e. absolute difference between first and second orientation). Subjects had to indicate as fast and accurately as possible whether the second orientation was tilted counterclockwise or clockwise relative to the first orientation by pressing a button. No feedback was provided to minimize feedback-based perceptual learning (Kahnt et al., 2011). Each trial type was repeated 7 times, resulting in a total of 70 trials. Discrimination performance was computed by averaging accuracy across trials and difficulty levels. The task was administered twice on each day: once immediately after administration of the pill to obtain a baseline measure in the absence of drug effects, and once after the scanning session (2 hr after drug/placebo administration) to measure discrimination performance under the influence of the drug (amisulpride plasma levels have a first peak after ~1 hr (Rosenzweig et al., 2002)). Due to technical problems, discrimination accuracy data from one subject were not saved.

### Comparing the kurtosis of behavioral generalization gradients

In order to compare the overall shape of the behavioral generalization gradients, we estimated the 4^th^ moment of the distributions underlying the behavioral gradients (because the behavioral gradients were bounded (17-–73 degrees) and not centered on 45 degrees, direct numerical estimation of the kurtosis was not possible). For this, we fitted a Pearson type VII distribution to the behavioral generalization gradient of each group and numerically computed the kurtosis of the group-specific distributions according to:

$$kurt\left(X\right)=\frac{\text{E}\left[{\left(X-\mu \right)}^{4}\right]}{\sigma^{4}}$$

The kurtosis was then compared between groups, and statistical inference on the observed group difference was performed using a permutation test.

### A computational model of similarity-based generalization

We designed a similarity-based generalization model (Figure 4A), based on previous computational approaches to stimulus generalization (Ghirlanda and Enquist, 1998; Kahnt et al., 2012; Pearce, 1987). The model assumes that each orientation *k* holds inhibitory and excitatory associations, *I_k_* and *E_k_* that change with learning. In a given trial *t*, the net associative strength *V* (or predicted reward) of the currently presented stimulus *k* equals the aggregated excitatory and inhibitory associative strengths of all stimuli *j* that that are generalized to stimulus *k* (including j = k):

$$V_{t}=\sum_{j}\;E_{t,j}\cdot eS_{j}^{k}-I_{t,j}\cdot iS_{j}^{k}$$

The degree to which associations generalize from stimulus *j* to *k* is determined by the inhibitory and excitatory generalization coefficients *iS_j_^k^* and *eS_j_^k^* (Figure 4B), respectively, which vary continuously between 0 and 1 (for *j* = *k*). These coefficients can take the form of Gaussians or exponential functions, and their widths (i.e. the width of generalization) are controlled by the parameters *s_i _*and *s_e_,* respectively:

$$iS_{j}^{k}=exp^{-\frac{{\left(x_{j}-x_{k}\right)}^{2}}{2\cdot s_{i}^{2}}}\;\text{and}\;eS_{j}^{k}=exp^{-\frac{{\left(x_{j}-x_{k}\right)}^{2}}{2\cdot s_{e}^{2}}}$$

Where *x_j_* and *x_k_* are the orientations (in degrees) of stimuli *j* and *k*, respectively. The corresponding exponential similarity functions are given by:

$$iS_{j}^{k}=exp^{{}^{-\frac{\left|x_{j}-x_{k}\right|}{2\cdot s_{i}^{2}}}}\;\text{and}\;eS_{j}^{k}=exp^{{}^{-\frac{\left|x_{j}-x_{k}\right|}{2\cdot s_{e}^{2}}}}$$

The excitatory and inhibitory strengths, *E* and *I*, are updated on every trial. Specifically, when the outcome *R* (1 or 0 for reward and no reward, respectively) is experienced, a prediction error *δ* is generated according to:

$$\delta_{t}=R-V_{t}$$

Because the prediction error is based on generalized reward predictions, it directly reflects the extent to which excitatory and inhibitory associations generalize to the currently presented stimulus. The prediction error is used to update the inhibitory and excitatory associative strengths according to:

$$E_{t+1,k}=E_{t,k}+\alpha \cdot \delta_{t}\;\;if\;\;\delta_{t}>0\;\;\text{and}\;\;\;\;I_{t+1,k}=I_{t,k}-\alpha \cdot \delta_{t}\;\;if\;\;\delta_{t}<0$$

where *α* is the learning rate. To account for different learning rates during discrimination training with feedback and generalization test without feedback, separate learning rates were allowed during training and test (*α_train_*, and *α_test_*).

The probability of making an approach response on a given trial *P(+)_t_* is given by the net associative strength *V_t_*, passed through a biasing sigmoid function (softmax), which is controlled by its slope *β* and offset *a*.

$$P{\left(+\right)}_{t}=\frac{1}{1+exp^{-\beta \cdot \left(V_{t}-a\right)}}$$

The free parameters of the model (*s_i_, s_e_, β, a, α_train_, α_test_*) were estimated by maximizing the log likelihood estimate (*LLE*) of subjects’ responses during test given the model

$$LLE=\underset{t}{\sum }\log P{\left(+|\theta \right)}_{t,}^{*}$$

where $P{\left(+|\theta \right)}_{t}^{*}$ is the probability of the model for making the same response as the subject in trial *t*. In order to compare models with Gaussian and exponential similarity functions, we estimated the free parameters of both models by combining the LLE from subjects in both groups, and compared the aggregate LLE from the best fitting parameter sets using AIC and BIC. In order to generate reliable parameter estimates for each group separately, the same fitting procedure was performed for each group (PA and PP) separately by combining *LLE* across all subjects within a group when evaluating the model. This yielded two sets of estimated model parameters (Table 1). In order to reduce the number of free parameters, and because both groups received placebo during training, the training learning rate (α_train_) was estimated based on data from all subjects, and then fixed to that value when estimating group-wise model parameters.

We tested the statistical significance of the observed differences in the group-wise model parameters using a permutation test. Specifically, we randomly assigned all subjects into one of the two groups, estimated a set of parameters for each group, and computed the difference between the estimated model parameters between groups. This was repeated 10,000 times, resulting in a distribution of group-wise parameter differences that should be expected by chance (i.e. if group assignments were random). This random distribution was then used to generate P-values for the empirically observed group-wise parameter differences.

To obtain an individual difference measure of model parameters, and as an alternative way to perform inference on the model parameters, we used a leave-one-out estimation procedure. For this, we first fitted the model using the data from all but one subject (N-1). The resulting model parameters were then subtracted from the parameters obtained when using all subjects (N). This difference (parameter based on N-1 subjects – parameter based on N subjects) is proportional to the relative contribution of the left-out subject to the entire group. For instance, if the left-out subject has a large “‘true”’ parameter, leaving this subject out when estimating the model will reduce the parameter obtained in the reduced sample (N-1) relative to the parameter obtained from the entire sample (N). In other words, this procedure generates estimates that reflect the individual differences in model parameters.

### fMRI data acquisition and preprocessing

Functional imaging was performed on a Philips Achieva 3 T whole-body scanner equipped with an eight-channel head coil. During the training and test sessions, 675 and 711 T2*-weighted whole-brain EPI images with 37 transversal slices were acquired with a repetition time (TR) of 2000 ms. Imaging parameters were as follows: slice thickness, 3 mm; in-plane resolution, 2.75 x 2.75 mm; echo time (TE), 30 ms; flip angle, 90°. For anatomical reference and identification of the dopaminergic midbrain, T1- and T2-weighted high-resolution (1 x 1 x 1 mm) anatomical images were acquired using the following imaging parameters. T1-weigthed: matrix size, 256 x 256; field of view, 256; 181 slices; flip angle, 8°; TR = 8.2 ms; TE = 3.8 ms. T2-weighted: matrix size, 256 x 256; field of view, 256; 181 slices; flip angle, 8°; TR = 2500 ms; TE = 248 ms. Preprocessing of functional images was performed using SPM12 and consisted of slice-time correction, realignment, coregistration of anatomical (T1-weighted) and functional images, spatial normalization to the standard template of the Montreal Neurological Institute (MNI) by estimating normalization parameters based on the T1-weighted image, and spatial smoothing using a Gaussian kernel of 8 mm FWHM.

### fMRI data analysis

To identify brain regions in which activity correlates with prediction errors derived from the similarity-based generalization model, we used a general linear model (GLM) with parametric modulators (Buchel et al., 1998) that included the following regressors: (1) onset of actual (training data) or expected (test data) time of outcome (offset of the response mapping screen), (2) a parametric modulator of stimulus orientation (z-standardized), and (3) a parametric modulator of prediction errors derived from the model with group-wise parameters (z-standardized). All regressors were convolved with a canonical hemodynamic response function (HRF) and together with the head movement parameters from the realignment procedure regressed against the BOLD signal in each voxel. Independent GLMs were estimated for the training and test session. Voxel-wise one-sample t-tests were applied to the resulting parameter estimates of the prediction error regressor.

To test for global effects of amisulpride on blood flow, and thereby BOLD response, we tested whether cue-evoked activity in visual cortex differed between groups. For this, we set up a GLM including one regressor for the onset of the visual cue (HRF convolved) and the six head movement parameters. Cue-related activity in an anatomical mask of the calcarine sulcus (AAL) did not differ between groups (t = -0.80, P = 0.42), suggesting that amisulpride did not unspecifically affect the BOLD response. This is in line with previous studies that found no differences in visually evoked activity between amisulpride and placebo (Jocham et al., 2011).

### Functional connectivity analysis

We examined dopamine-related differences in the functional connectivity between the midbrain and the hippocampus by using a variant of the psycho-physiological interaction (PPI) model (McLaren et al., 2012). For each subject, the average time course was extracted from voxels in a 50% probabilistic mask of the substantia nigra (Murty et al., 2014), and together with the six head movement parameters regressed against the time course in each voxel. The parameter estimate of the midbrain-seed regressor reflects the correlation between activity in the midbrain and activity in every other voxel in the brain. To identify regions where connectivity differed depending on D2R blockade, the connectivity maps were compared between groups using a two-sample t test.

### Statistical analysis

To identify significant voxels in the fMRI analysis (prediction error during test or training), we used one-sample t-tests and a threshold of P < 0.05, FWE whole brain corrected, in combination with a cluster extent threshold of k>10. To test for group differences, parameter estimates were extracted from significant voxels in the striatum (training) and hippocampus (test), and compared between groups using two-sample t-tests at a statistical threshold of P < 0.05. Significant group differences in functional connectivity were identified using a threshold of P < 0.05, FWE small volume corrected for a functional region of interest in the hippocampus that was identified in the independent prediction error contrast during test in the entire group of subjects (P < 0.05, FWE-corrected). A priori comparisons using t-tests and correlations with directed hypotheses are tested one-tailed.

Although amisulpride is generally known to not alter RT (Jocham et al., 2011; Rosenzweig et al., 2002), we found a trend-level group difference in RT (t = 1.83, P = 0.074), with slower responding in amisulpride (mean RT ± SEM = 816 ± 16.6) compared to placebo (mean RT ± SEM = 770 ± 18.5). Importantly, all group comparisons remained significant when RT was included as a covariate in the statistical models, suggesting that non-significant differences in RT did not confound the imaging results.
