# Supplementary material for: Dopamine regulates stimulus generalization in the human hippocampus
Source: eLife. 2016 Feb 2;5:e12678. doi: 10.7554/eLife.12678 (PMC4755747; doi:10.7554/eLife.12678)
Supplement: Related texts

# Article commentary: Decision letter

**DOI:** 10.7554/eLife.12678.013

**Affiliations:** Brown University, United States

eLife posts the editorial decision letter and author response on a selection of the published articles (subject to the approval of the authors). An edited version of the letter sent to the authors after peer review is shown, indicating the substantive concerns or comments; minor concerns are not usually shown. Reviewers have the opportunity to discuss the decision before the letter is sent (see review process). Similarly, the author response typically shows only responses to the major concerns raised by the reviewers.

[Editors’ note: a previous version of this study was rejected after peer review, but the authors submitted for reconsideration. The first decision letter after peer review is shown below.]

Thank you for choosing to send your work entitled "Dopamine controls stimulus generalization in the human hippocampus" for consideration at *eLife*. Your full submission has been evaluated by Timothy Behrens (Senior Editor), Michael Frank (Reviewing Editor), and two peer reviewers. Based on our discussions and the individual reviews below, we regret to inform you that your work will not be considered further for publication in *eLife* in its current form.

All reviewers and editors expressed interest in the findings and framework, but questioned the level of advance over and above what your group has published in 2012 with a similar model and relation to hippocampal-striatal connectivity. The main issues that came up in the review discussion (some of which are reiterated by the individual reviewer comments below) are as follows.

– First and foremost, while the dopaminergic effect is of real potential interest, all were concerned that there was no observable effect of the drug on generalization behavior, but only through the lens of the model parameters. In general a model can certainly be helpful to refine behavioral analysis, but (1) it has to be shown to fit the data well before its parameters can be interpreted, (2) it should be compared to alternative potential models, and (3) if both of these are successful the winning model should then guide an analysis of the behavioral data that would reveal the significant effects. Reviewers were also concerned that other (unmodelled) factors (e.g. new learning during testing session affected by dopamine) could be at play which make it less clear that dopamine is specifically changing the hippocampal generalization gradient, and that the current findings weren't well integrated with your previous findings.

– The specificity of the effects was not fully established. Reviewers suggest control analyses that would address whether indeed what was seen in the hippocampus is something that is unique to the hippocampus, or whether it was a general effect of pharmacology in BOLD signal that was not observed elsewhere.

*Reviewer #1:* This study examined the role of dopamine in modulation of perceptual generalization, combining pharmacology, fMRI and computational modeling.

On the first day participants trained on a visual discrimination task. On the second day they were tested for generalization of the training to new samples varying in similarity to the trained items. The generalization test took place either under drug (D2 receptor blocker) or under placebo, in a between-subject design. The results suggest that both groups generalize similarly, overall. But the application of a computational model of generalization revealed subtle but interesting differences in the width of the generalization gradient, with a narrower gradient in the drug-treated group. This difference in the generalization gradient was associated with differences in BOLD activity in the hippocampus, with the drug group showing a weaker generalization-related BOLD response.

Overall this is a solid and informative study using a convergence of methods to address an interesting question. I have just a few comments:

1) As with any pharmacological study, one must raise concerns about selectivity. How do you know the effects of the drug are not due to global differences in dopamine transmission, that are having an impact on BOLD activity in multiple places in parallel with (but not necessarily related to) the subtle differences in behavior? The study already addresses this somewhat by showing selectivity of effects on functional connectivity between the hippocampus and the midbrain, but not with the striatum. And the findings ruling out mere differences in perception also help address this point. But similar control analyses are also needed for the generalization gradient, to determine whether there is really a selective effect of the drug in the hippocampus, as the authors currently conclude. For example, Paz and colleagues have suggested an important role for the amygdala and the PFC in perceptual generalization. These regions are also targets of midbrain region and it would be useful to know whether or not they show parallel effects.

– Related to the point about possible global differences between drug and placebo, were there any reaction time differences between the groups?

2) The Discussion makes the important distinction between associative and perceptual generalization, but this distinction is muddled in the introduction where the two appear to be conflated. It will help readers if this distinction is clarified earlier on.

*Reviewer #2:* The study by Kahnt and Tobler investigates the role of dopamine in stimulus generalization using fMRI, computational modeling and dopamine blockade (D2R). Two groups of subjects first learnt which of two oriented gabor patches (CS+ 39 deg, CS- 51) were associated with positive or neutral outcome. Following drug (PA) or placebo (PP) administration they then performed the generalization test where they responded to 15 different orientations not presented during training (between 17-73 deg; no outcome presented) with multiple repetitions of each item. Main findings of interest: i) as in their (2012) and other studies a classic peak shift generalization curve was observed (i.e. away from CS-) ii) whilst there was no significant difference between the groups in the raw data, groups different when a similarity based model of generalization was used (i.e. excitatory generalization coefficient was larger in PP group; n.s. trend for inhib coefficient). Iii) Modelled prediction errors during generalization were observed in the hippocampus, and greater in the PP group. Further there was a reduction in midbrain-hippocampus functional connectivity in the PA group, that was associated with individual differences in modeled inhibitory coefficient. Overall findings are discussed as implicating dopaminergic circuit and hippocampus in flexible stimulus generalization based on neuromodulatory state.

This study addresses a question of importance and the findings are potentially of interest. However, I have some concerns about the interpretation of the findings (see below). In addition I am not sure whether the current paper represents a substantial enough advance above their published 2012 work (where an analogous computational model was used and interactions between the hippocampus-striatum implicated) to warrant publication in *eLife*. The main novel aspect is the dopaminergic manipulation, but as I see it there are some inconsistencies between the 2012 and this paper which make it difficult to have a coherent picture.

Major concerns:

Given the effect of drug on generalization curve is relatively small (i.e. only detectable by model fitting), it seems important that the authors consider alternative models. If these also showed a significant difference in group parameters this would make the findings appear more robust. In particular, exemplar models (e.g. Nosofsky, 1984) and models of similarity based generalization (e.g Shepard, 1987) typically use an exponential similarity function. Was there a particular reason for opting for a Gaussian function instead, and could the authors please provide results from an analogous model with exponential function for comparison? This would be particularly useful given the possible relationship between exemplar models and the hippocampus.

One important claim of the paper is that dopamine widens the generalization curve (e.g. modeled by increase in excitatory coefficient relating to width of Gaussian). Whilst this makes sense at face value, I wonder whether that there are potentially complicated interactions at play during the generalization test that may (also/instead) be affected by dopamine. In brief, and detailed subsequently: dopamine is known to affect the strength of hippocampal encoding and perhaps this is the reason for changes in the generalization curve. This would be important (apart from changing the mechanistic explanation) because it suggests that the dopamine/hippocampal effect is to some extent paradigm specific: it does not apply to "one-shot" generalization (perhaps more naturalistic), but only under settings where lots of stimuli are presented and generalization required.

To state this in more detail: subjects are learning during the generalization phase: i.e. storing new representations of the previously unseen test items and their predicted values (which are being constantly updated). It certainly seems conceivable that in this setting the hippocampus is important in setting up these representations, and the behavioral generalization curve then reflects the concurrent retrieval of these representations in parallel (i.e. as in exemplar models) to compute an EV for the current stimulus (as the authors suggest). My question is how a difference in the strength of these item representations in the hippocampus could influence the generalization curve? At present this is not modeled, and given the known role of dopaminergic modulation of hippocampal encoding this seems an additional factor to consider. Assuming the authors agree with this, could they incorporate this as an additional parameter in the model (distinct from the learning rate, which applies to value updating), and report the results?

It seems important for the authors relate these findings more closely to those of their previous study, which is not discussed in detail at present and consider possible inconsistencies. Firstly, no hippocampal PE signals were reported in that study: can the authors comment on any reasons for why this might be the case (e.g. paradigm differences etc? Secondly, stronger hippocampal-striatal connectivity in that study was associated with narrower generalization: here stronger midbrain-hippocampal connectivity was associated with broader generalization. Can the authors comment on the difference in findings, and also whether the midbrain-hippocampal connectivity result is replicable in the original dataset?

Hippocampal activity is reported to correlate with generalized PE during test phase. Given that no outcomes are presented, is the PE not equivalent to EV? If so, it would be worth stating this, partly because in recent years a number of studies have found EV to be reflected in hippocampal activity.

[Editors’ note: what now follows is the decision letter after the authors submitted for further consideration.]

Thank you for resubmitting your work entitled "Dopamine regulates stimulus generalization in the human hippocampus" for further consideration at *eLife*. Your revised article has been favorably evaluated by Timothy Behrens (Senior Editor), Michael Frank (Reviewing Editor), and two reviewers. We all agreed that the manuscript has been substantially improved and that you have done a nice job addressing the main issues. Still, there are a few remaining issues that need to be addressed before acceptance, as outlined below by each reviewer in turn.

I highlight two points here.

First, both Reviewers appreciated the new analyses showing specificity of the effects to the hippocampus, but each would like to see a follow-up analysis to further evaluate this specificity. Reviewer 1 notes that any strong claims regarding specificity need to show a region by drug effect interaction and not just a significant effect in hippocampus and null effect elsewhere (see e.g., Nieuwenhuis, Forstmann & Waenmakkers 2011 Nature Neuroscience who emphasize this point strongly). Reviewer 2 asks whether you also see specificity in the PPI analysis.

Second, Reviewer 2 would like to see a somewhat more fleshed out motivation for the prediction error analysis and to clarify whether the PE was evaluated at the outcome or during the choice.

Please address these and the other comments below, and we will be able to make a decision without further review.

*Reviewer #1:* This is a resubmission of a previous paper relating to the role of the hippocampus & dopamine in stimulus generalization. Overall, the authors have been very responsive to the concerns I had with the original paper: I was convinced by the new findings that distinguish the effect of drug in the absence of a model (through differences in kurtosis), the results of the inclusion of a similarity based model with exponential function, and consideration of learning during test as a possible explanatory factor. Also the discussion of the current findings with respect to their previous paper is informative.

I have few remaining concerns:

– The authors report a significant difference between drug/placebo groups in terms of prediction error signals in the hippocampus, but report that this effect was not significant in other regions showing prediction error signals (e.g. amygdala). Can they confirm whether there was a significant interaction (i.e. drug/placebo x brain region), for example in an appropriately constructed region of interest analysis? This seems important to demonstrate the specificity of the drug effect on PE signals.

– They observe that midbrain-hippocampal functional connectivity correlates with the inhibitory (but not excitatory) generalization coefficient of the model. Although there is a comment that relates to this in the Discussion, could the authors expand on why the behavioral findings between drug groups should be driven by differences in the excitatory coefficient, but not the functional connectivity findings? Also was there a significant difference between placebo and drug group in this correlation?

*Reviewer #2:* The authors did an extensive revision in which they addressed the main concerns raised in the last round, particularly the lack of a behavioral difference under drug and the lack of specificity of the fMRI findings. The result is an interesting paper presenting solid findings that are likely to be of broad interest.

I have a couple of remaining comments:

1) The rational for the prediction error analysis in the test phase is not spelled out clearly enough. Perhaps I misunderstood, but I thought the authors were arguing that the effect of dopamine at test is essentially on the retrieval of a learned representation, the generalization itself, and that it is explicitly not related to trial by trial updating during the test phase. This leaves me questioning what the link is to the prediction error at the outcome phase rather than the response/choice at the stimulus presentation phase.

2) The selectivity of the fMRI effects to the hippocampus are reassuring. What about the PPI effects? Here too it seems important to show regional selectivity, ideally using the same control regions, to show that the PPI differences are not generally related to pharmacological effects on BOLD.

# Reply: Author response

**DOI:** 10.7554/eLife.12678.014

[Editors’ note: the author responses to the first round of peer review follow.]

We have now thoroughly revised the manuscript based on these comments. Specifically, we provide evidence for an effect of amisulpride on generalization behavior by showing significant group differences in the shape (i.e., kurtosis) of the behavioral generalization gradients (see reviewer #2, point 1). We also show that the effect of amisulpride on model parameters controlling the width of generalization does not depend on the choice of the specific model (see reviewer #2, point 1). We have now compared our model to alternative models, including a model with an exponential similarity function and a model with additional sensory learning during test (see reviewer #2, point 2). We demonstrate that a Gaussian similarity function explains the data better than an exponential similarity function and that additional learning during test cannot account for our results. Importantly, we now show that the effects of dopamine on generalization related fMRI signals are specific to the hippocampus and do not extend into other regions involved in generalization such as the amygdala and the mPFC (see reviewer #1, point 1). We also clarify the conceptual advances over our previous study (Kahnt et al. 2012, J Neurosci), provided by using a pharmacological manipulation of dopamine, and a dissociation of encoding- and retrieval-based generalization (see reviewer #2, point 3).

All reviewers and editors expressed interest in the findings and framework, but questioned the level of advance over and above what your group has published in 2012 with a similar model and relation to hippocampal-striatal connectivity. The main issues that came up in the review discussion (some of which are reiterated by the individual reviewer comments below) are as follows. – First and foremost, while the dopaminergic effect is of real potential interest, all were concerned that there was no observable effect of the drug on generalization behavior, but only through the lens of the model parameters. In general a model can certainly be helpful to refine behavioral analysis, but (1) it has to be shown to fit the data well before its parameters can be interpreted, (2) it should be compared to alternative potential models, and (3) if both of these are successful the winning model should then guide an analysis of the behavioral data that would reveal the significant effects. Reviewers were also concerned that other (unmodelled) factors (e.g. new learning during testing session affected by dopamine) could be at play which make it less clear that dopamine is specifically changing the hippocampal generalization gradient, and that the current findings weren't well integrated with your previous findings. – The specificity of the effects was not fully established. Reviewers suggest control analyses that would address whether indeed what was seen in the hippocampus is something that is unique to the hippocampus, or whether it was a general effect of pharmacology in BOLD signal that was not observed elsewhere.

We have now revised the manuscript in light of these comments and believe that they have strengthened the manuscript considerably. Specifically, we now demonstrate an effect of amisulpride on the shape of behavioral generalization gradients by showing that compared to placebo, gradients in the amisulpride group have a significantly greater kurtosis. We also show that our model fits the behavioral data well, and compare our model to alternative potential models. Specifically, we demonstrate that the effect of amisulpride on model parameters controlling the width of generalization does not depend on the choice of the specific model. Moreover, we show that our original model with a Gaussian similarity function outperforms a model with an exponential similarity function and that adding a sensory learning mechanism does not improve the fit of the model. We then use the winning model to guide the analysis of the behavioral and neural data and characterize significant effects of the dopamine manipulation on model parameters. We also show that additional learning during test does not explain our results.

Importantly, we show that the effects of dopamine on generalization related fMRI signals are specific to the hippocampus and do not occur in other regions such as the amygdala, middle temporal gyrus and mPFC.

We also clarify the conceptual advancements over our previous study (Kahnt et al. 2012, J Neurosci), provided by using a dopamine receptor type-specific manipulation as opposed to a simple correlation between brain activity and behavior, and the dissociation of encoding- and retrieval-based generalization. The point-by-point responses below describe in detail how we addressed each of the reviewers’ comments.

Reviewer #1:

This study examined the role of dopamine in modulation of perceptual generalization, combining pharmacology, fMRI and computational modeling.

*On the first day participants trained on a visual discrimination task. On the second day they were tested for generalization of the training to new samples varying in similarity to the trained items. The generalization test took place either under drug (D2 receptor blocker) or under placebo, in a between-subject design. The results suggest that both groups generalize similarly, overall. But the application of a computational model of generalization revealed subtle but interesting differences in the width of the generalization gradient, with a narrower gradient in the drug-treated group. This difference in the generalization gradient was associated with differences in BOLD activity in the hippocampus, with the drug group showing a weaker generalization-related BOLD response. Overall this is a solid and informative study using a convergence of methods to address an interesting question. I have just a few comments: 1) As with any pharmacological study, one must raise concerns about selectivity. How do you know the effects of the drug are not due to global differences in dopamine transmission, that are having an impact on BOLD activity in multiple places in parallel with (but not necessarily related to) the subtle differences in behavior? The study already addresses this somewhat by showing selectivity of effects on functional connectivity between the hippocampus and the midbrain, but not with the striatum. And the findings ruling out mere differences in perception also help address this point. But similar control analyses are also needed for the generalization gradient, to determine whether there is really a selective effect of the drug in the hippocampus, as the authors currently conclude. For example, Paz and colleagues have suggested an important role for the amygdala and the PFC in perceptual generalization. These regions are also targets of midbrain region and it would be useful to know whether or not they show parallel effects.*

We thank the reviewer for raising this important point. There are two potential sources of non-selectivity that need to be considered:

1) First, there could be pharmacological effects on global blood flow that are independent of dopaminergic activity. Because the early visual cortex contains only very few dopamine receptors (Lidow et al. 1989, PNAS), visual activity provides a good control for non-specific (i.e., non-dopaminergic) effects of the drug on blood flow and the BOLD response. We therefore tested for drug-related differences in visual cue-related activity in the early visual cortex (AAL, calcarine sulcus). We did not find any differences in cue-related activity in early visual cortex between groups (t = -0.80, P = 0.42), suggesting that amisulpride did not affect global blood flow. This is in line with previous studies that found no differences between amisulpride and placebo in visually evoked activity (e.g., Jocham, et al. 2011, J Neurosci).

2) Second, because we delivered amisulpride systemically rather than by intracerebral infusion, it could have altered BOLD responses in all brain regions that contain dopamine receptors (such as the amygdala), independent of their role in generalization. In other words, the question is whether the observed effects of amisulpride on hippocampal activity are unique to the hippocampus, or can also be identified in other dopaminoceptive brain regions that are involved in generalization. As a control, we therefore tested whether generalization-related activity in the amygdala and middle temporal cortex was also altered by amisulpride. We did not find significant differences in generalization-related activity in either the amygdala (P = 0.43) or the middle temporal gyrus (P = 0.31), suggesting that the effects of amisulpride on generalization-related activity are specific to the hippocampus.

While we do not find generalization-related activity in the PFC at our corrected threshold, we do find a cluster in the medial PFC (mPFC, x, y, z = -3, 56, 8, t = 4.03) at a more liberal, uncorrected threshold (P < 0.001). In order to test for potential drug effects in the PFC, we compared activity in this cluster across groups. The comparison between groups did not reveal any differences (t = -0.023, P = 0.98), further supporting the specificity of the hippocampus finding. Because the mPFC finding is not based on a corrected, but a more liberal uncorrected threshold, we decided to not include this finding in the manuscript. However, we would be happy to include these results if the reviewer feels this would strengthen the manuscript.

Taken together, the results from the two control analyses demonstrate that our findings cannot be explained by non-specific drug effects on blood flow, and that the effects of dopamine on generalization-related processing are specific to the hippocampus. We now include the results of these control analyses in the manuscript:

Methods:

“To test for global effects of amisulpride on blood flow, and thereby BOLD response, we tested whether cue-evoked activity in visual cortex differed between groups. For this, we set up a GLM including one regressor for the onset of the visual cue (HRF convolved) and the six head movement parameters. Cue-related activity in an anatomical mask of the calcarine sulcus (AAL) did not differ between groups (t = -0.80, P = 0.42), suggesting that amisulpride did not unspecifically affect the BOLD response. This is in line with previous studies that found no differences between amisulpride and placebo in visually evoked activity (Jocham et al., 2011).”

Results:

“To examine whether these effects of dopamine on generalization-related activity are specific to the hippocampus, as a control, we tested for similar group differences in the amygdala and middle temporal cortex. Importantly, we did not find significant differences in either the amygdala (P = 0.43) or the middle temporal gyrus (P = 0.31), in line with the idea that the effects of amisulpride on generalization-related activity are specific to the hippocampus.“

*– Related to the point about possible global differences between drug and placebo, were there any reaction time differences between the groups?*

As suggested by the reviewer, we tested for group differences in response time (RT). Although amisulpride is generally known not to alter RT (e.g., Rosenzweig 2002, Hum Psychopharmacol Clin Exp; Jocham et al. 2011, J Neurosci), we found a non-significant group difference in RT (t = 1.83, P = 0.074), with slower responding in amisulpride (mean RT +/- SEM = 816 +/- 16.6) compared to placebo (mean RT +/- SEM = 770 +/- 18.5). All group comparisons remained significant when RT was included as a covariate in the statistical models, suggesting that non-significant differences in RT did not confound the imaging results. We now add this to the manuscript.

Methods:

“Although amisulpride is generally known to not alter RT (Jocham et al., 2011; Rosenzweig et al., 2002), we found a trend-level group difference in RT (t = 1.83, P = 0.074), with slower responding in amisulpride (mean RT ± SEM = 816 ± 16.6) compared to placebo (mean RT ± SEM = 770 ± 18.5). Importantly, all group comparisons remained significant when RT was included as a covariate in the statistical models, suggesting that non-significant differences in RT did not confound the imaging results.”

*2) The Discussion makes the important distinction between associative and perceptual generalization, but this distinction is muddled in the introduction where the two appear to be conflated. It will help readers if this distinction is clarified earlier on.*

We now explicitly mention this distinction in the Introduction.

Introduction: “Two basic forms of generalization can be distinguished based on what constitutes the relation among stimuli; associative and stimulus generalization. In the case of associative generalization, such as transitive inference and acquired equivalence, the associative relationship among stimuli determines similarity. This relationship can be established for example by sensory preconditioning (train stimuli A-B and B-C, test whether A comes to predict C) or by a common associate (e.g. train A-C and B-C, test whether A and B are associated). In contrast, in stimulus generalization the relationship among stimuli is established by the similarity along one or more perceptual dimensions (frequency of sounds, color, line orientation, etc.).“

Reviewer #2:

*Major concerns: Given the effect of drug on generalization curve is relatively small (i.e. only detectable by model fitting), it seems important that the authors consider alternative models. If these also showed a significant difference in group parameters this would make the findings appear more robust. In particular, exemplar models (e.g. Nosofsky, 1984) and models of similarity based generalization (e.g Shepard, 1987) typically use an exponential similarity function. Was there a particular reason for opting for a Gaussian function instead, and could the authors please provide results from an analogous model with exponential function for comparison? This would be particularly useful given the possible relationship between exemplar models and the hippocampus.*

Thank you for raising this important point. We now report evidence for an effect of amisulpride on the shape of the observed behavioral generalization gradients independent of a specific computational model. Specifically, while none of the individual data points along the generalization gradient individually differed between groups, overall, gradients in the amisulpride group were reduced at both flanks and enhanced at the peak and tail of the curve. These features of the shape of the gradients are parsimoniously captured by the kurtosis of the underlying distributions. We therefore compared the kurtosis of the group-specific distributions (Pearson type VII distribution), and found significantly greater kurtosis in the distribution of the amisulpride group (permutation test, P = 0.043). This result provides evidence for effects of the drug on the overall shape of the behavioral generalization gradients. We now include this in the manuscript.

Abstract:

“Blocking dopamine D2-receptors (D2R) altered generalization behavior as revealed by an increased kurtosis of the generalization gradient, and a decreased width of model-derived generalization parameters.”

Results:

“Direct group comparisons of the individual data points along the generalization gradient did not reveal any significant differences (two-sample t-tests, all Ps > 0.29). However, visual inspection of the gradients suggested that the amisulpride group had a narrower gradient than the placebo group, with enhanced responding at the peak of the curve, reduced responding at both flanks, and enhanced responding at the tail of the curve. These shape features are parsimoniously described by the 4th moment of probability distributions, namely, their kurtosis. Accordingly, a test for differences in the kurtosis of group-specific distributions (Pearson type VII distribution, see Materials and methods) revealed a significantly greater kurtosis in the amisulpride group compared to placebo (PA: 6.73, PP: 3.29; permutation test, P = 0.043). This finding demonstrates that amisulpride narrowed the width and increased the peak of the behavioral generalization gradient, and suggests that D2R activity alters the neurocomputational processes that mechanistically control generalization behavior.”

Materials and methods: “Comparing the kurtosis of behavioral generalization gradients”.: “In order to compare the overall shape of the behavioral generalization gradients, we estimated the 4th moment of the distributions underlying the behavioral gradients (because the behavioral gradients were bounded (17-73 degrees) and not centered on 45 degrees, direct numerical estimation of the kurtosis was not possible). For this, we fitted a Pearson type VII distribution to the behavioral generalization gradient of each group and numerically computed the kurtosis of the group-specific distributions according to:

$$kurt\left(X\right)=\frac{\text{E}\left[{\left(X-\mu \right)}^{4}\right]}{\sigma^{4}}$$

The kurtosis was then compared between groups, and statistical inference on the observed group difference was performed using a permutation test.”

Regarding the shape of the modeled similarity function (i.e., generalization coefficient), we note that Shepard (1987) indeed proposed that similarity decreases exponentially with distance from the CS. However, he also listed several factors, such as repeated discrimination training and delays between training and test (both apply to our current experiment), that can render similarity functions Gaussian. Accordingly, and in line with the results of systematic reviews of empirical data (Ghirlanda & Enquist, 2003, Animal Behaviour), we initially opted for a Gaussian similarity function for modeling the behavior.

To assess the validity of our choice, and as suggested by the reviewer, we formally compared models with Gaussian and exponential similarity functions. The exponential similarity functions for the inhibitory and excitatory gradients were implemented as:

$iS_{j}^{k}=exp^{-\frac{{\left(x_{j}-x_{k}\right)}^{2}}{2∙s_{i}^{2}}}$and$eS_{j}^{k}=exp^{-\frac{{\left(x_{j}-x_{k}\right)}^{2}}{2∙s_{e}^{2}}}$As can be seen in Figure 2–figure supplement 1, compared to the Gaussian model, the exponential model was substantially worse in capturing the behavioral generalization gradients of both groups. Also, the exponential model was unable to capture the group differences in the shapes of the generalization gradients, and accordingly, the estimated generalization coefficients did not differ between groups (inhibitory coefficient, P = 0.34, excitatory coefficient, P = 0.24). A formal model comparison showed that the model with the Gaussian similarity function outperformed the model with the exponential similarity function as indicated by smaller AIC and BIC scores (Gaussian: AIC = 9586.4, BIC = 9629.2; Exponential: AIC = 9691.6, BIC = 9734.4). We also compared the fit of the two models by comparing the average regression coefficients from a logistic regression of the trial-by-trial responses on the modeled P(+) responses. This revealed significantly better fits of the Gaussian compared to the exponential model in the entire group of subjects (paired t-test t = 5.47, P < 0.001), as well as in both groups individually (PA, t = 3.34, P = 0.003 and PP, t = 5.72, P < 0.001).

Taken together, these findings are in line with the observations by Shepard (1987) and demonstrate that the Gaussian model fits our data better than a model with an exponential similarity function. As suggested by the Editors, we now address this point by including a formal model comparison before testing for group differences in the estimated parameters of the winning (i.e., Gaussian) model.

Results:

“Because the shape of the function determining the similarity between the currently presented stimulus and other stimuli (i.e., the generalization coefficient) is of critical importance (Ghirlanda and Enquist, 2003), we directly compared the most commonly used models, i.e. one with Gaussian (Kahnt et al., 2012) the other with exponential similarity functions (Shepard, 1987). While the exact shape of the similarity functions differs between models, for both models, the extent to which inhibitory and excitatory associations generalize to the current stimulus is controlled by the width of the similarity functions (si and se) (Figure 4B). […] Although both models predicted behavioral responses reliably (Gaussian: t = 11.06, P < 0.001; exponential: t = 11.38, P < 0.001), we find significantly higher regression coefficients for the Gaussian model (paired t-test t = 5.34, P < 0.001). Taken together, this demonstrates that in our experiment, a Gaussian similarity function fits behavior better than an exponential similarity function. “

Materials and methods:

“In order to compare models with Gaussian and exponential similarity functions, we estimated the free parameters of both models by combining the LLE from subjects in both groups, and compared the aggregate LLE from the best fitting parameter sets using AIC and BIC.”

It is important to note that the effect of amisulpride on the model parameters controlling the width of generalization does not depend on the a priori choice of the Gaussian model. Specifically, the difference between the exponential and Gaussian similarity functions is determined by the exponent of the (absolute) difference between xj and xk (1 and 2 for the exponential and Gaussian model, respectively). Instead of comparing the fit of different theoretical shapes (exponential and Gaussian), we have also directly estimated the best fitting exponent by including the exponent as an additional free parameter in the model. This resulted in a parameter of 2.04, close to the assumed Gaussian model. Also for this model, the width of the inhibitory and excitatory generalization coefficients differed significantly between groups (P = 0.013 and P = 0.016, for the inhibitory and excitatory coefficients, respectively). However, because this exponential model came at the expense of an additional parameter, without substantially increasing the LLE, it was outperformed by the Gaussian model as indicated by lower AIC and BIC values for the Gaussian model (AIC = 9586.4 vs. 9588.3 and BIC = 9629.2 vs. 9638.2 for Gaussian vs. exponent as free parameter, respectively). These results demonstrate that the effect of amisulpride on the parameters controlling the width of generalization does not depend on the choice of the specific model. In the interest of parsimony, we tentatively opted against including this model in the manuscript. However, if the reviewer feels this should be included, we would be happy to revise the manuscript accordingly.

*One important claim of the paper is that dopamine widens the generalization curve (e.g. modeled by increase in excitatory coefficient relating to width of Gaussian). Whilst this makes sense at face value, I wonder whether that there are potentially complicated interactions at play during the generalization test that may (also/instead) be affected by dopamine. In brief, and detailed subsequently: dopamine is known to affect the strength of hippocampal encoding and perhaps this is the reason for changes in the generalization curve. This would be important (apart from changing the mechanistic explanation) because it suggests that the dopamine/hippocampal effect is to some extent paradigm specific: it does not apply to "one-shot" generalization (perhaps more naturalistic), but only under settings where lots of stimuli are presented and generalization required. To state this in more detail: subjects are learning during the generalization phase: i.e. storing new representations of the previously unseen test items and their predicted values (which are being constantly updated). It certainly seems conceivable that in this setting the hippocampus is important in setting up these representations, and the behavioral generalization curve then reflects the concurrent retrieval of these representations in parallel (i.e. as in exemplar models) to compute an EV for the current stimulus (as the authors suggest). My question is how a difference in the strength of these item representations in the hippocampus could influence the generalization curve? At present this is not modeled, and given the known role of dopaminergic modulation of hippocampal encoding this seems an additional factor to consider. Assuming the authors agree with this, could they incorporate this as an additional parameter in the model (distinct from the learning rate, which applies to value updating), and report the results?*

The reviewer addresses an interesting point here, namely that dopamine-related differences in learning during the test session could have affected the shape of the generalization gradient. It is important to point out that our original model already accounts for additional associative learning during test. That is, test stimuli acquire stimulus-outcome associations by means of the same learning mechanism that is used during training (δ rule). The estimated learning rates for value learning during test (α_test_) did not differ between groups (P = 0.214), and therefore cannot account for differences in the shape of generalization gradients between groups.

If we understand correctly, the reviewer proposes that dopamine could affect a different form of learning during test, instead of, or in addition to, value learning. Specifically, it is possible that dopamine alters the sensory encoding of novel test stimuli for which further associative learning takes place. Such sensory representations could gradually build up over the test session as test stimuli are repeatedly encountered. In this scenario, test stimuli can contribute to generalized expected value (V) only to the degree that sensory representations for these stimuli actually have been established.

To explore this possibility, we modeled the gradual encoding of test stimuli using a mnemonic stimulus buffer M. Before a given test stimulus x_k_ is encountered for the first time, this buffer is zero, and is then updated on a trial by trial basis using the δ rule and a sensory learning rate (α_sensory_):

$$\Delta M_{k}{=}_{sensory}∙{\left(1-M_{k}\right)}_{}$$

Please note that a sensory learning rate of 1 (α_sensory_ = 1) renders the model equivalent to our original model, in which sensory representations of test stimuli (M) are fully established after the first encounter.

When the generalized expected value (V) is computed on a given trial, M serves to discount the contribution of the test stimuli’s excitatory and inhibitory associations (I_k_ and E_k_):

$V_{t}=\underset{j}{\sum }M_{k}∙E_{t,j}∙eS_{j}^{k}-M_{k}∙I_{t,j}∙iS_{j}^{k}$As in our original model, the excitatory and inhibitory associations of the test stimuli (I_k_ and E_k_) are updated using the value learning rate (α_test_):

and $$E_{t+1,k}=E_{t,k}+\alpha ∙\delta_{t}if\delta_{t}>0$$

$I_{t+1,k}=I_{t,k}-\alpha ∙\delta_{t}if\delta_{t}<0$With $\delta_{t}=R-V_{t}$Fitting α_sensory_ for the entire group of subjects revealed an α_sensory_ of 1, demonstrating that the fit of our initial model was not enhanced by adding the gradual sensory encoding mechanism. Accordingly, because of the additional free parameter, both AIC and BIC were in favor of our original model (AIC = 9588.4 vs. 9586.4; and BIC = 9638.3 vs. 9629.2).

Moreover, estimating the model in both groups separately also revealed an α_sensory_ of 1 in both groups, suggesting that dopamine did not affect how sensory representations of test stimuli are encoded over the course of the test session.

It is worth noting that the two learning rates α_sensory_ and α_test_ are not entirely independent. Specifically, within certain ranges (i.e., α_sensory_ > 0 and α_test_ > 0), changes in α_sensory_ can be compensated by changes in α_test_, and vice versa. This is because across test trials the δ rule for the update of I_k_ and E_k_ similarly induces a gradually increasing contribution of individual test stimuli to the generalized value prediction V. Nevertheless, the fact that both learning rates did not differ between groups indicates that additional learning during the test session was not altered by dopamine, and can therefore not account for the differences in generalization gradients.

In summary, these analyses suggest that our results cannot be explained by effects of dopamine on the encoding of sensory stimulus representations during test. Even though this is an interesting possibility, for the sake of clarity, we tentatively opted to not include the above results in the manuscript. However, if the reviewer feels that this should be included, we would be happy to incorporate these passages.

We now state explicitly that additional learning during the test session did not differ between groups.

Results:

“Importantly, the learning rate during test did not differ between groups, demonstrating that additional learning during the test session (P = 0.21), which might have been altered by amisulpride, cannot account for the differences in generalization gradients.”

*It seems important for the authors relate these findings more closely to those of their previous study, which is not discussed in detail at present and consider possible inconsistencies. Firstly, no hippocampal PE signals were reported in that study: can the authors comment on any reasons for why this might be the case (e.g. paradigm differences etc? Secondly, stronger hippocampal-striatal connectivity in that study was associated with narrower generalization: here stronger midbrain-hippocampal connectivity was associated with broader generalization. Can the authors comment on the difference in findings, and also whether the midbrain-hippocampal connectivity result is replicable in the original dataset?*

We now describe the differences between our previous (2012) and the current study more clearly. Most importantly, our previous study was silent about the pharmacology and timing of stimulus generalization. Procedurally, it consisted of several alternating training and testing sessions on a single day, whereas the current study used a single training and a single test session, which were separated by a 24 h delay. Moreover, in the 2012 study CS-US associations were deterministic, whereas the current study involved a 50% reinforcement schedule in order to slow down extinction during test. These factors could have caused the differences in PE related activity in the striatum vs. hippocampus. However, it is worth stating that in the 2012 data we did observe PE related activity in the hippocampus (left [-36, -25, -8], t = 3.84; right [30, -7, -14], t = 3.97, P < 0.001), but these activations did not survive correction for multiple comparisons and were therefore not reported in the final manuscript.

Moreover, in the 2012 study, hippocampal-striatal connectivity was negatively correlated with the width of the excitatory vs. inhibitory gradients (excitatory minus inhibitory). As such, this negative correlation could have been driven by a negative correlation with the excitatory gradient, and/or a positive correlation with the inhibitory gradient. In fact, going back to the original data, we find only a relatively weak negative correlation between striatum-hippocampus connectivity and the excitatory gradient (r = -0.42, P = 0.053), but a strong and positive correlation between connectivity and the inhibitory gradient (r = 0.57, P = 0.005). This shows that the relationship between striatal-hippocampal connectivity and the inhibitory gradient in the previous study is compatible with the results reported here.

We also revisited the data from the 2012 study with regard to the current finding of a positive correlation between midbrain-hippocampal connectivity and the width of the inhibitory coefficient. Replicating the results of our current study, we find that midbrain-hippocampal connectivity was significantly correlated with the inhibitory generalization coefficient (r = 0.44, P = 0.036), but not with the excitatory coefficient (r = 0.15, P = 0.49).

We now discuss the differences to our previous study, and relate the findings more thoroughly.

Discussion:

“By revealing the effects of dopamine on generalization, our current results substantially extend those of our previous study (Kahnt et al., 2012). Specifically, the current experiment suggests that dopamine is involved in regulating the width of generalization in the hippocampus, and dissociates effects of generalization during retrieval vs. encoding, which could not be achieved with the previous design. However, whereas here we find that prediction errors correlate primarily with activity in the hippocampus, the previous study identified prediction error related activity primarily in the ventral striatum. […] Thus, the two studies converge in showing a preferential relation of the connection between the midbrain and the hippocampus to inhibitory generalization.”

*Hippocampal activity is reported to correlate with generalized PE during test phase. Given that no outcomes are presented, is the PE not equivalent to EV? If so, it would be worth stating this, partly because in recent years a number of studies have found EV to be reflected in hippocampal activity.*

The reviewer is correct that because no outcomes were shown in the test session, PEs are perfectly (but negatively) correlated with EV. We now mention this in the paper and include references reporting value signals in the hippocampus.

Results:

“Please note that because no outcomes were shown, prediction errors are perfectly (but negatively) correlated with expected value.”

[Editors' note: the author responses to the re-review follow.]

*First, both Reviewers appreciated the new analyses showing specificity of the effects to the hippocampus, but each would like to see a follow-up analysis to further evaluate this specificity. Reviewer 1 notes that any strong claims regarding specificity need to show a region by drug effect interaction and not just a significant effect in hippocampus and null effect elsewhere (see e.g., Nieuwenhuis, Forstmann & Waenmakkers 2011 Nature Neuroscience who emphasize this point strongly). Reviewer 2 asks whether you also see specificity in the PPI analysis. Second, Reviewer 2 would like to see a somewhat more fleshed out motivation for the prediction error analysis and to clarify whether the PE was evaluated at the outcome or during the choice.*

We thank the editors for the positive evaluation of our resubmitted manuscript. Below we address the remaining comments. Most importantly, we now include explicit tests of drug-by-region interactions to formally evaluate the specificity of the drug effects in the hippocampus. We also address the remaining interpretative issues.

Reviewer #1:

*This is a resubmission of a previous paper relating to the role of the hippocampus & dopamine in stimulus generalization. Overall, the authors have been very responsive to the concerns I had with the original paper: I was convinced by the new findings that distinguish the effect of drug in the absence of a model (through differences in kurtosis), the results of the inclusion of a similarity based model with exponential function, and consideration of learning during test as a possible explanatory factor. Also the discussion of the current findings with respect to their previous paper is informative. I have few remaining concerns:*

We thank the reviewer for these positive comments on the resubmitted version of our manuscript. Below we address the remaining comments.

*– The authors report a significant difference between drug/placebo groups in terms of prediction error signals in the hippocampus, but report that this effect was not significant in other regions showing prediction error signals (e.g. amygdala). Can they confirm whether there was a significant interaction (i.e. drug/placebo x brain region), for example in an appropriately constructed region of interest analysis? This seems important to demonstrate the specificity of the drug effect on PE signals.*

We agree with the reviewer that in order to demonstrate specificity, interactions need to be computed. In line with specificity, post-hoc analyses comparing the effect of the drug in the hippocampus to the drug effect in the other regions (i.e., group-by-region interactions), demonstrated that the drug effect in the hippocampus was significantly stronger than in the mPFC (P = 0.02). However, similar interactions involving the amygdala (P = 0.097) and the middle temporal gyrus (P = 0.127) did not reach significance. This suggests that while the drug effects are specific to the hippocampus relative to the mPFC, they do not exceed the non-significant drug effects in the amygdala and the middle temporal gyrus. We now add these results to the manuscript and adjust our interpretation regarding specificity accordingly.

Results:

“No significant group differences were observed in the amygdala (P = 0.43), the middle temporal gyrus (P = 0.31), or the medial PFC (P = 0.98). However, post-hoc analyses directly comparing the effect of the drug in the hippocampus to the drug effect in the other regions (i.e., region-by-group interactions), demonstrated that while the effect of D2R blockade in the hippocampus was significantly stronger than in the mPFC (P = 0.02) similar interactions involving the amygdala (P = 0.097) and the middle temporal gyrus (P = 0.127) did not reach significance. These data suggest specificity of the effects of D2R blockade on similarity-based processing in the hippocampus relative to the mPFC, but not necessarily relative to the amygdala and middle temporal lobe.”

*– They observe that midbrain-hippocampal functional connectivity correlates with the inhibitory (but not excitatory) generalization coefficient of the model. Although there is a comment that relates to this in the Discussion, could the authors expand on why the behavioral findings between drug groups should be driven by differences in the excitatory coefficient, but not the functional connectivity findings? Also was there a significant difference between placebo and drug group in this correlation?*

One possibility is that the decrease in the inhibitory and the excitatory coefficient induced by D2R blockade relies on separate neural mechanisms. Specifically, it is possible that only the effects of D2R blockade on the inhibitory coefficient are mediated by connectivity between midbrain and the hippocampus, whereas the D2R effects on excitatory coefficient are mediated via a different mechanism. The difference in the correlation between the placebo and the drug group was only at trend level (p = 0.054). We now spell out this possibility more explicitly in the revised manuscript:

Discussion:

“This raises the possibility that only the effects of D2R blockade on the inhibitory coefficient are mediated via a modulation of midbrain-hippocampal connectivity, whereas the effects on the excitatory coefficient are mediated via a different, yet to be explored, mechanism.”

Reviewer #2:

*The authors did an extensive revision in which they addressed the main concerns raised in the last round, particularly the lack of a behavioral difference under drug and the lack of specificity of the fMRI findings. The result is an interesting paper presenting solid findings that are likely to be of broad interest. I have a couple of remaining comments: 1) The rational for the prediction error analysis in the test phase is not spelled out clearly enough. Perhaps I misunderstood, but I thought the authors were arguing that the effect of dopamine at test is essentially on the retrieval of a learned representation, the generalization itself, and that it is explicitly not related to trial by trial updating during the test phase. This leaves me questioning what the link is to the prediction error at the outcome phase rather than the response/choice at the stimulus presentation phase.*

The reviewer is right, our results do suggest that the effect of dopamine is on the retrieval of learned information. Because no outcomes are shown during the test phase, we can use prediction errors as a proxy for generalized retrieved value. The prediction error was modeled at the time of outcome. However, we note that because of the fast timing of our task, it is difficult to dissociate effects during the stimulus/choice period from that of the outcome. We now make this more explicit in the manuscript.

Results:

“As a proxy of generalized value, we focused on prediction error responses derived from our model, which reflect the extent to which reward predictions have generalized from the original CS+ and CS− to the current stimulus (please note that because no outcomes were shown, prediction errors are perfectly but negatively correlated with expected value). Accordingly, to identify brain regions involved in similarity-based computations during generalization, we searched for regions in which fMRI activity correlated with generalized prediction errors at the time of the expected outcome.”

*2) The selectivity of the fMRI effects to the hippocampus are reassuring. What about the PPI effects? Here too it seems important to show regional selectivity, ideally using the same control regions, to show that the PPI differences are not generally related to pharmacological effects on BOLD.*

We examined the specificity of the connectivity effects and now include these results in the manuscript.

Results:

“To examine the specificity of these findings, we tested for similar drug-related effects on functional connectivity in the regions involved in similarity-based processing defined above. While connectivity estimates differed significantly between groups in middle temporal gyrus (P = 0.01), no drug effects were observed in the amygdala (P = 0.296) and the mPFC (P = 0.17). Accordingly, for all regions except the middle temporal gyrus (P = 0.13), the corresponding drug-by-region interactions were significant (all Ps < 0.05), suggesting that amisulpride-related decreases in midbrain connectivity are relatively specific to the hippocampus.”
